# Re-Programming Autoreactive T Cells Into T-Regulatory Type 1 Cells for the Treatment of Autoimmunity

**DOI:** 10.3389/fimmu.2021.684240

**Published:** 2021-07-15

**Authors:** Patricia Solé, Pere Santamaria

**Affiliations:** ^1^ Institut D’Investigacions Biomèdiques August Pi i Sunyer, Barcelona, Spain; ^2^ Julia McFarlane Diabetes Research Centre (JMDRC) and Department of Microbiology, Immunology and Infectious Diseases, Snyder Institute for Chronic Diseases and Hotchkiss Brain Institute, Cumming School of Medicine, University of Calgary, Calgary, AB, Canada

**Keywords:** T-regulatory type 1 (TR1) cells, peptide-MHC class II-coated nanoparticles, T-cell reprogramming, interleukin 10 (IL10), autoimmune disease, therapy

## Abstract

Systemic delivery of peptide-major histocompatibility complex (pMHC) class II-based nanomedicines can re-program cognate autoantigen-experienced CD4+ T cells into disease-suppressing T-regulatory type 1 (TR1)-like cells. In turn, these TR1-like cells trigger the formation of complex regulatory cell networks that can effectively suppress organ-specific autoimmunity without impairing normal immunity. In this review, we summarize our current understanding of the transcriptional, phenotypic and functional make up of TR1-like cells as described in the literature. The true identity and direct precursors of these cells remain unclear, in particular whether TR1-like cells comprise a single terminally-differentiated lymphocyte population with distinct transcriptional and epigenetic features, or a collection of phenotypically different subsets sharing key regulatory properties. We propose that detailed transcriptional and epigenetic characterization of homogeneous pools of TR1-like cells will unravel this conundrum.

## Introduction

Interleukin 10 (IL-10)-producing regulatory T cells (Tregs) are key to immune homeostasis and play opposing roles in autoimmunity versus cancer. While the FoxP3+ Treg cell subset has been thoroughly described, FoxP3 and CD25 double-negative T cells producing IL-10 in the context of low IL-4 secretion are generally known as T-regulatory type 1 (TR1) cells (1). Given the lack of specificity of these phenotypic descriptors, the literature has considered as TR1-like cells what appears to be a rather heterogeneous collection of cell types (1), thus clouding our understanding of the true lineage identity of this regulatory T-cell subset. Production of IL-10, coupled to the expression of Latency-Associated Peptide (LAP), Lymphocyte Activation Gene 3 (LAG-3) or CCR5 and Programmed cell death protein 1 (PD-1) in the absence of CD25, or CD4+ cells lacking IL-7R expression, as well as cells induced by vitamin D3 or CD46-stimulation are some of the examples of cell types identified as TR1 (1, 2). Recently, co-expression of CD49b and LAG-3, accompanied by the expression of ICOS and PD-1, has been associated, in both humans and mice, with TR1-ness (3, 4), but these markers are not sufficiently specific or sensitive. Other surface markers have been found to be variably upregulated by IL-10-producing T-cell subsets (5–8), including Cytotoxic T-Lymphocyte antigen 4 (CTLA-4), T-cell immunoglobulin and mucin-domain containing-3 (TIM-3) or TIGIT, as well as transcription factors (TFs) like T-bet, Aryl hydrocarbon receptor (AhR) or Nuclear Factor Interleukin 3-regulated (Nfil3).

Because of the lack of specific markers, it remains unclear whether the various IL-10 producing ‘TR1-like’ subsets correspond to multiple different cell types, or to cells at different stages of differentiation. Many studies implicating a role for Treg/TR1 cells in the therapeutic activity of various immunotherapies have often done so solely based on an increase in IL-10 expression by splenic CD4+ T cells. It is entirely possible that the various phenotypes associated to IL-10-producing FoxP3-negative CD4+ T-cell subsets correspond to cells at different stages of TR1 cell differentiation, or to distinct subsets of terminally differentiated cells with distinct phenotypic and/or functional properties. Unfortunately, the transcriptional and epigenetic profiles associated with true TR1-ness remain incompletely defined, a fact compounded by our incomplete knowledge on the developmental biology of the TR1 subset(s).

**Figure 1:**
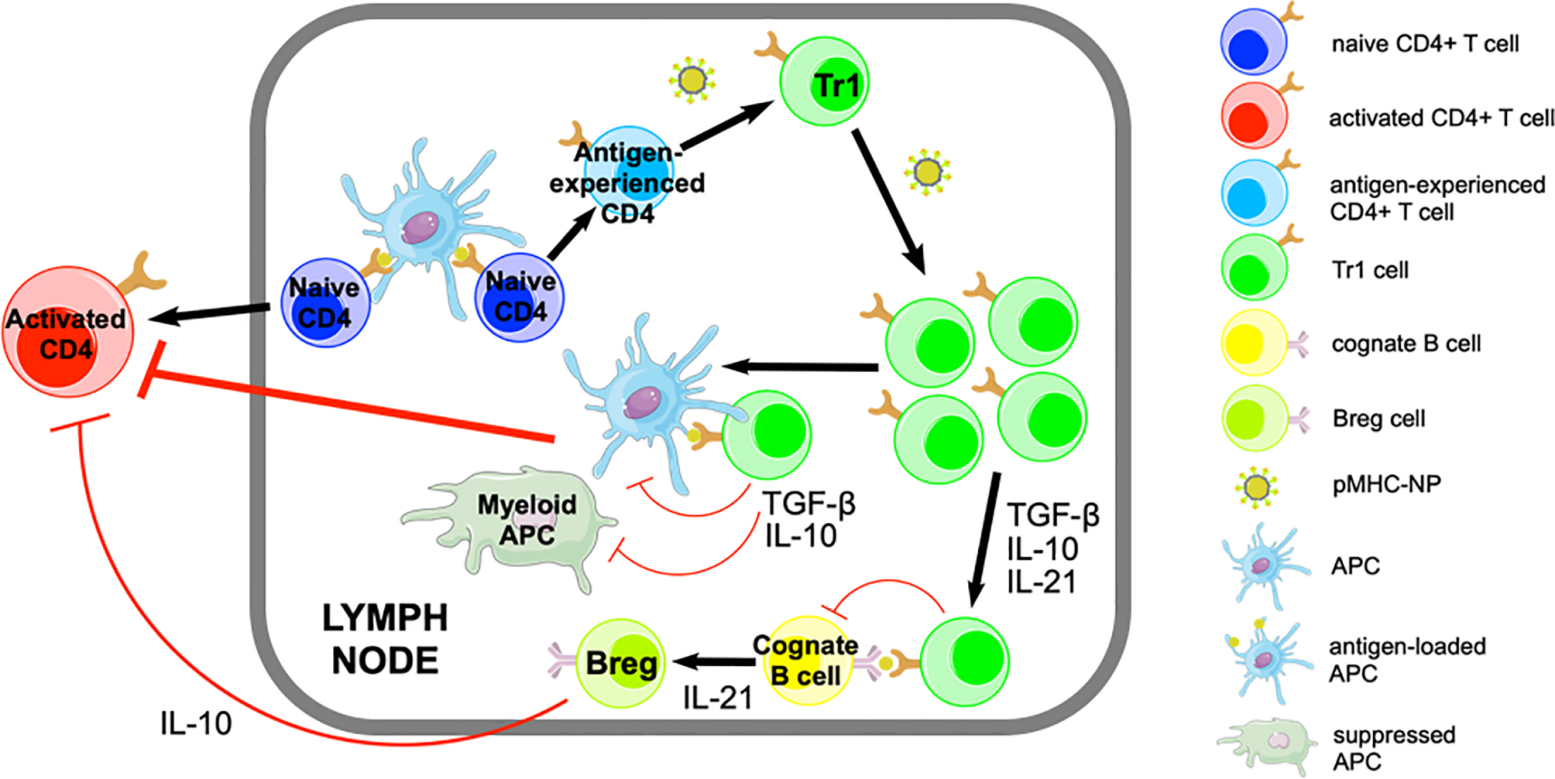
Pharmacodynamic activity of pMHCII-NPs. pMHCII-NPs target autoantigen-experienced CD4+ T cells and induce their differentiation into memory TR1-like cells followed by their systemic expansion. This process involves IFN-γ and IL-10 signaling, but does not require IL-27. pMHCII-NP-induced TR1-like cells carry out their regulatory function by suppressing other autoreactive T-cell specificities *via* IL-10, IL-21 and TGF-β. IL-10 and TGF-β have immunosuppressive effects on autoantigen-loaded APCs, inhibiting their proinflammatory function and thus avoiding the activation of other non-cognate autoreactive T cells. pMHCII-NP-induced TR1-like cells can also interact with cognate B-cells, promoting their differentiation into Bregs in part *via* IL-21. Figure adapted from Clemente-Casares et al. (11).

We have shown that treatment of various mouse models of autoimmune disease with nanoparticles (NPs) coated with disease-relevant peptide-major histocompatibility complex class II (pMHCII) molecules (9) suppresses organ inflammation and disease progression without impairing systemic immunity (10–12). This approach has shown clear therapeutic efficacy in animal models of type 1 diabetes (T1D), experimental autoimmune encephalomyelitis (EAE), collagen-induced arthritis (11), as well as primary biliary cholangitis (PBC), primary sclerosing cholangitis (PSC) and autoimmune hepatitis (AIH) (12, 13). pMHCII-NP therapy triggers the formation and expansion of TR1-like CD4+ T cells from autoantigen-experienced CD4+ T-cell precursors of as yet undefined identity. pMHCII-NPs bind directly to TCRs on cognate T cells, resulting in prolonged pMHCII-TCR interactions, the assembly of large TCR microclusters on such T cells, and rapid, robust and prolonged TCR signaling. In turn, this results in the acquisition of immunoregulatory properties, including the upregulation of the cytokines IL-10, IL-21 and Transforming Growth Factor β (TGF-β) (but neither IL-2 nor IL-4), the co-inhibitory receptors LAG-3, CTLA-4 and PD-1, the Inducible T-cell Costimulator (ICOS) and the transcription factors T-bet and c-Maf, among others, in the absence of FoxP3 expression (**Figure 1**).

Here, we review our current understanding of the phenotype, function and development of TR1-like cells in different experimental settings, including pMHCII-NP-treated mice. We identify knowledge gaps and propose that detailed transcriptional and epigenetic characterization of homogeneous pools of TR1-like cells will help define both, a true state of TR1-ness as well as the identity of the TR1-poised cell precursors that give rise to TR1-like cells.

## A Brief Historical Perspective

The TR1 cell subset was first described in 1997 by Groux et al. (14). Previously, others (15, 16) had described a suppressor T-cell population that secreted IL-10 and protected patients against graft-versus-host disease (GvHD). This population displayed a cytokine profile that was distinct from those of common T-helper cell subsets, and involved the expression of IL-10, IL-5, TGF-β, and IFN-γ in the absence of IL-4 or IL-2 secretion (17). In 1997, TR1 cells were generated *in vitro* and their suppressive activity was documented both *in vitro* and *in vivo*, in a model of colitis.

Currently, all the regulatory CD4+ T cells that are FoxP3-negative and secrete IL-10 and low levels or no IL-4 are considered to be ‘TR1’. Unfortunately, this characterization lacks specificity and likely includes phenotypically, functionally and developmentally heterogeneous T cells. This is compounded by the variety of protocols that can trigger the formation of IL-10-producing cells with regulatory properties. In some cases, TR1-like cells were generated from naive CD4+ T cells. For example, *in vitro* TCR stimulation of human naive CD4+/CD45RA+ T cells in the context of IL-10 secreted by dendritic cells (DCs) triggered their conversion into anergic, IL-10- and TGF-β-expressing T cells capable of suppressing effector T cells (14, 18). Likewise, *in vitro* culture of murine CD4+/CD44–/CD62L+ T cells with IL-10 or IL-27 can induce their differentiation into IL-10 producing TR1-like cells [reviewed in (19)]. Other lines of experimentation have suggested that IL-10-producing TR1-like cells can also be generated from memory CD4+ T cells, in the absence of polarizing cytokines in the culture (20, 21). In mice, induction of transplantation tolerance *via* anti-CD45RB mAb therapy is associated with the presence of antigen-specific IL-10-producing CD4+ T cells in the memory T-cell compartment (22, 23). There are also data supporting the view that TR1-like cells can develop from differentiated T-helper cell subsets. For example, Gagliani et al. provided evidence suggesting that a fraction of the regulatory T cells that are found in the gut arise from Th17 cells and display a TR1-like phenotype, including the production of IL-10 and some IFN-γ, and the expression of CD49b and LAG-3, while lacking expression of IL-4 and CCR6 (24). Moreover, there is also evidence that culture of Th17 cells in the presence of IL-27 and TGF-β can trigger the formation of IL-10-producing TR1-like cells *in vitro* (24, 25). Likewise, stimulation of Th1 cells with CXCL12 *in vitro* (26), or in the context of malaria infection (27), can promote their differentiation into CD4+/CD25–/FoxP3–/IL-10+ T cells. Human allergen-specific Th2 cells can also differentiate into IL-10-producing CD49b+/LAG3+ cells with regulatory properties (28, 29).

Unfortunately, these various ‘TR1-like’ cell types of different developmental origin were not thoroughly characterized at the phenotypic, transcriptional or functional levels. Accordingly, whether the various TR1-like cells that were generated in these studies correspond to one or several different cell types, or to cells at different stages of differentiation, remains unclear.

## Distinct Phenotypes

Several surface phenotypes have been attributed to TR1-like cells (**Table 1**). Whether all these subsets correspond to one single, incompletely characterized population, or comprise a collection of phenotypically and/or functionally distinct subsets remains to be determined.

**Table 1:** Summary of phenotypes ascribed to TR1-like cells.

*Markers*	*Where*	*Phenotype*	*Species*	*Reference*
*LAP+/CD25–/CD4+*	~3-5% of murine splenocytes	High levels of TGF-β and IL-10, IL-2, IL-4 and IFN-γ	Mouse	(30)
	After oral anti-CD3 treatment	Suppressive effect in autoimmune encephalomyelitis	Mouse	(31)
*NKG2D+/CD25–/CD4+*	In peripheral blood of healthy individuals (~1-3%). Increased in cancer (~6-70%)		Human	(32)
	In patients with rheumatoid arthritis		Human	(33)
*CD127^low^/CD25–/CD4+*	~1% of CD4+ of human PBMCs	Low levels of Bcl-2 and high levels of Ki-67 and ICOS Secretion of IL-10 upon TCR engagement	Human	(34)
*CD49b+/CD25–/CD4+*	In mice	Secretion of IL-10 TGF-β and IFN-γ. Anti-diabetogenic and anti-arthritogenic	Mouse	(35–38)
*LAG-3+/CD25–/CD4+*	In the spleen (2%), lymph nodes (1%) and Peyer’s patches (PP) (8%)	Anergic upon TCR ligation, secrete IL-10 and IFN-γ, and low amounts of IL-2 and IL-4. Expression of Egr-2 and Blimp-1	Human	(39)
*CD49b+/LAG-3+/ CD25–/CD4+*	Peripheral blood	IL-10 producing suppressive cells	Human/ mouse	(3)
*CCR5+/PD-1+/ CD25–/CD4+*	Lamina propria	Secretion of IL-10- and IFN-γ. Expression of LAG-3 upon stimulation	Human	(2, 40)
*CD44^hi^/CD62L^lo^/IL-7R–/LAG-3+/CD49b+/ LAP+*	Spleen and draining lymph nodes of pMHCII-NP-treated mice	Secretion of IL-10, IL-21, TGF-β and IFN-γ, but no IL-2, IL-4 or IL-17. Expression of c-Maf, T-bet and Blimp-1.	Mouse	(11)
***Other markers***				
*TIGIT*			Mouse	(5)
*TIM-3*			Mouse	(8)
*CD226*			Human/ mouse	(3, 41)
*ROG*			Mouse	(42)
*Egr-2*			Mouse	(43)
*c-Maf and AhR*	IL-27-induced TR1-like cells		Mouse	(44, 45)
*IRF4*	Activin-A stimulated human TR1-like cells		Human	(46)
*LXR*			Human	(7)
*Bhlhe40*			Human	(7)

### LAP+/CD25–/CD4+ T Cells

CD4+ CD25+ Treg cells express TGF-β on their surface and one of their mechanisms of suppression involves TGF-β recognition by target cells upon cell-to-cell contact (47). Weiner et al. reported a population of regulatory T cells that suppressed murine colitis in a TGF-β-dependent manner, but where CD25-negative and LAP-positive (30). LAP is the amino-terminal domain of the TGF-β precursor peptide that contains the TGF-β peptide within its latent complex (48). CD4+/CD25–/LAP+ cells are positive for thrombospondin, which can convert latent TGF-β to its active form. CD4+/CD25–/LAP+ cells represent ~3-5% of murine splenocytes and express high levels of TGF-β and IL-10, as well as IL-2, IL-4 and IFN-γ. A similar population was generated after oral anti-CD3 treatment and had a suppressive effect against autoimmune encephalomyelitis (31).

### NKG2D+/CD25–/CD4+ T Cells

A small population of human CD4+ T cells that produce IL-10 and TGF-β express the natural killer receptor NKG2D. These cells are FoxP3-, CD103- and LAG-3-negative. They also express Fas ligand (FasL), which appears to be a main contributor of suppression by inhibiting the growth of bystander T cells (32). Although these T cells can be found in the peripheral blood of healthy individuals (~1-3%), they appear to increase substantially in cancer patients (to ~6-70%). They have also been described in patients with rheumatoid arthritis (33). One ligand of the NKG2D receptor is the MHC class I-related chain A (MICA), which is upregulated in tissues undergoing inflammation or in epithelial tumors. The role of NKG2D with regards to the immunoregulatory properties of these cells remains unclear.

### CD127^low^/CD25–/CD4+ T Cells

The IL-7 receptor (IL-7R) α-chain (CD127) is important for the survival of conventional CD4+ T cells (49) but is expressed at low levels in CD4+CD25+ T cells (50). Häringer et al. found a population of adaptive Treg cells that were CD25-, FoxP3- and IL-7R-negative. These cells comprised ~1% of the total CD4+ population from human peripheral blood. They expressed low levels of Bcl-2 and high levels of Ki-67 and ICOS, suggesting that they had been recently activated, and had a suppressive function mediated primarily by the secretion of IL-10 in response to potent T-cell receptor stimuli (34). However, only 10% of this T-cell pool produced IL-10 upon stimulation, compatible with the presence of a small subset of TR1-like cells within the CD25–/FoxP3–/IL-7R– pool.

### CD49b+/CD25–/CD4+ T Cells

Several studies have identified a population of CD4+ T cells with regulatory activity that express CD49b. These cells had anti-diabetogenic (35) and anti-arthritogenic properties in mice (36), were both FoxP3– and CD25– and secreted the regulatory cytokines IL-10 and TGF-β, as well as IFN-γ. In later studies, it was shown that these T cells suppressed CD8+ T-cell responses and IFN-γ production by CD4+ T cells, presumably *via* IL-10 (37, 38).

### LAG-3+/CD25–/CD4+ T Cells

LAG-3 is known to suppress T-cell proliferation (51). Despite being required for the maximal regulatory activity of conventional CD4+CD25+ Treg cells, LAG-3 protein can hardly be detected on the surface of CD4+CD25+ T cells. In contrast, LAG-3 was found to be expressed by a subset of CD4+CD25– T cells (39) found at low frequencies in the spleen (2%) and lymph nodes (1%) but at higher frequencies in Peyer’s patches (PP) (8%). These T cells are anergic upon TCR ligation, but they secrete high quantities of IL-10, moderate amounts of IFN-γ and low amounts of IL-2 and IL-4. These cells do not express FoxP3 and, unlike CD4+/CD25–/LAP+ cells, express low levels of CD103 and LAP. They are further characterized by expression of the Early response gene 2 (Egr-2), a transcription factor that is a negative regulator of T-cell activation, inducing an anergic state (52). These CD4+/CD25–/LAG-3+ cells were also found to express the *Prdm1* gene, encoding the B lymphocyte-induced maturation protein (Blimp)-1.

### CD49b+/LAG-3+/CD25–/CD4+ T Cells

In 2013, Gagliani et al. provided evidence indicating that co-expression of LAG-3 and CD49b can be used to enumerate human and mouse TR1-like cells (3). CD49b had been previously described as a marker for regulatory CD25– T cells, but cannot be used in isolation to identify this T-cell subset, because it can also be expressed by Th17 cells and certain memory CD4+ T-cell subsets (53). Likewise, LAG-3 is associated with T-cell activation and IL-10 production, but its expression is not unique to any particular T-cell subset; it can be upregulated by conventional T cells upon activation and is also expressed by FoxP3+ Tregs (51).

### CCR5+/PD-1+/CD25–/CD4+ T Cells

Geginat and coworkers used the C-C chemokine receptor type 5 (CCR5) and PD-1 as markers to purify TR1-like IL-10- and IFN-γ-producing cells from the human intestine (2, 40). They demonstrated that the majority of IL-10+/CD4+/CD25–/IL-7R– T cells found in the lamina propria co-expressed CCR5+ and PD-1+ (2). Despite expressing *Lag3* mRNA, only a small percentage of cells displayed LAG-3 protein in the steady state. *In vitro* stimulation triggered the upregulation of surface LAG-3 protein expression (2).

### Other Markers

TR1-like cells express several other surface molecules and transcription factors, albeit none of them specifically. For example, both murine and human IL-10 producing TR1-like cells can express the immune checkpoint molecules TIGIT (5) and TIM-3 (8), but conventional FoxP3+ Treg cells and T-follicular regulatory (Tfr) cells (54, 55) can also express these markers. CD226, presumably involved in the cytotoxic activity of at least some TR1-like cells, is another example of such lack of specificity (3, 41).

With regards to transcription factors, ROG (the repressor of GATA-3), a regulator of Th differentiation and cytokine production upon activation (56), has also been described in TR1-like cells (42). Since expression of Egr-2 in CD4+ T cells induces IL-10 production by binding to the Blimp-1 promoter (57), Okamura et al. proposed that this transcription factor might be involved in the acquisition of a suppressor phenotype by CD4+/CD25–/LAG-3+ T cells (43). However, purified TR1-like cells from the gut of anti-CD3 mAb-treated mice, as well as those induced *in vitro*, express levels of Egr-2 that are no different than those seen in effector T cells (3). Likewise, the transcription factors c-Maf and AhR, which are expressed by IL-27-induced TR1 cells and bind to the *Il10* promoter in TR1 cells (44, 45), are also expressed by non-TR1 cell types, including human and murine Th17 subsets (58, 59). The interferon regulatory factor 4 (IRF4) is yet another non-TR1 cell-specific transcription factor that presumably plays a role in the developmental biology of TR1-like cells, as a downstream effector of the inducible tyrosine kinase (ITK) (60). Since IRF4 regulates Blimp-1, it is probably involved in the regulation of IL-10 expression in these cells, along with other transcription factors. Activin A-induced IRF4 activation has been suggested to promote human TR1-like cell formation *in vitro* (46). The liver X receptor (LXR) and Bhlhe40 are other transcription factors found to be expressed in at least some TR1-like cells (7).

### pMHCII-NP-Induced TR1-Like Cells

When compared to other TR1-like subsets, the IL-10-producing CD44^hi^/CD62L^lo^/IL-7R–/CD25–/FoxP3– TR1-like cells that arise *in vivo* in response to pMHCII-NP therapy co-express several of the markers previously identified in different TR1-like cell subsets, including LAG-3, CD49b, ICOS, LAP, c-Maf, T-bet, and Blimp-1. These cells produce the cytokines IL-10, IL-21 and, to a lesser extent, IFN-γ, but no or very low levels of IL-2, IL-4 or IL-17 (11). Thus, these cells appear to embody the phenotypic properties of most other TR1-like cells, begging the question of whether different IL-10-expressing CD4+CD25– TR1-like cell subsets, as described in the literature, correspond to one single cell subset rather than to a phenotypically heterogenous collection of distinct cell types.

## Mechanisms of Action

**Figure 2:**
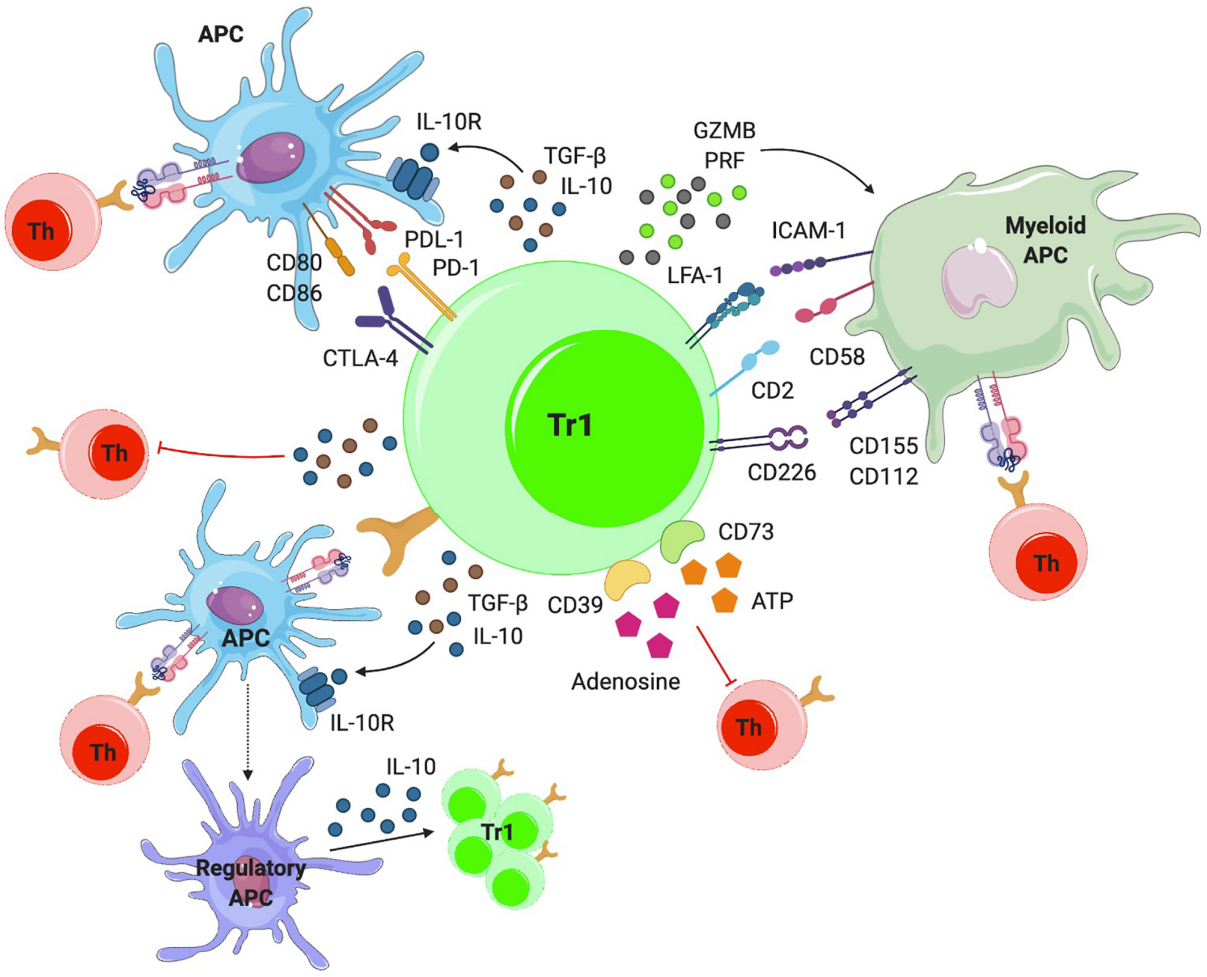
Mechanisms of action. TR1-like cells can suppress effector T cells directly or indirectly, by modulating the phenotype and function of APCs *via* IL-10 and TGF-β. IL-10 also induces a regulatory phenotype in APCs, by triggering the upregulation of tolerogenic molecules (i.e. ILT3, ILT4 and HLA-G) and the production of IL-10, further amplifying TR1-like cell formation. TR1-like cells can also make direct cell-to-cell contacts with APCs *via* cell surface CTLA-4 and PD-1, inhibiting APC-induced effector T-cell activation. TR1-like cells can kill myeloid APCs *via* granzyme-B (GZMB) and perforin (PRF). In addition, they can suppress T-cell activation *via* metabolic disruption.

In order to affect regulatory activity, TR1 cells need to be activated by antigen recognition. Upon activation, they target effector T cells and/or professional APCs *via* cytokines, direct cell contact, metabolic disruption and/or cytolysis (**Figure 2**). Although TCR activation is antigen-specific, TR1-mediated suppression of APCs or neighboring T cells is antigen-agnostic (bystander immunoregulation).

### Interleukin 10

Upon activation, TR1 cells secrete the immunoregulatory cytokines IL-10 and TGF-β (**Figure 2**). IL-10 has effects on different cell populations. Although IL-10 expression is a hallmark of TR1-like cells, this cytokine can also be produced by other CD4+ T-cell subsets, as well as CD8+ T cells, macrophages, DCs and B cells (61). IL-10 suppresses T-cell responses by inhibiting T-cell proliferation (62) and cytokine production by effector T cells, including IL-2, IFN-γ, IL-4, IL-5 and TNF-α. Moreover, IL-10 can downregulate MHC class II and costimulatory molecule expression in APCs, and reduce the production of pro-inflammatory cytokines (IL-1α and -β, IL-6, IL-12, IL-18, and TNF-α) and chemokines (CCL2, CCL5, CCL12, CXCL2, CXCL10, and IL-8) by these APCs (61). In humans, IL-10 can elicit the generation of tolerogenic DCs by upregulating immunoglobulin-like transcripts 3 and 4 (ILT3, ILT4) and the non-classical HLA-G molecule (63). On B-cells, IL-10 promotes proliferation, MHC II expression and isotype switching to IgG4 (64). IL-10 also amplifies regulatory T-cell formation. IL-10 stimulation of CD4+ T cells can induce the expression of IL-10, T-cell anergy or TR1-like cell differentiation in a STAT3-dependent manner. STAT3 promotes IL-10 expression and represses pro-inflammatory cytokine expression (65). It is unclear whether the phenotype of full-fledged (i.e. fully differentiated) TR1-like cells is stable. However, pMHCII-NP-induced, antigen-specific TR1-like cells can persist for several months post-treatment withdrawal without any obvious loss of key phenotypic properties or acquisition of pathogenic activity (11).

### Transforming Growth Factor β

Like IL-10, TGF-β inhibits APC function and T-cell proliferation, differentiation and cytokine production (**Figure 2**). TGF-β suppresses T-cell proliferation by inhibiting IL-2 production and downregulating cyclins while upregulating cyclin-dependent kinase (CDK) inhibitors. It also inhibits the differentiation of both CD4+ and CD8+ T cells into effectors, by inhibiting master transcriptional regulators of each phenotype (GATA-3, T-bet, IL-12Rβ2). The main effect of TGF-β on APCs involves inhibition of their maturation, in part by upregulating indoleamine 2,3-dioxygenase (IDO) expression and by inhibiting MyD88-mediated TLR signaling (66). As shown in (11, 12), the therapeutic effects of pMHCII-NP-induced TR1-like cells are dependent on IL-10 and TGF-β. The blockade of these cytokines with monoclonal antibodies abrogates the suppression of autoantigen crosspresentation by pMHCII-NP-expanded TR1-like cells and thus the therapeutic properties of pMHCII-NP treatment in several models, including T1D, EAE and liver autoimmunity.

### Costimulatory and Co-Inhibitory Molecules

TR1-like cells can also inhibit APCs in a cell contact-dependent manner upon engagement of co-inhibitory receptors such as CTLA-4, PD-1, LAG-3 or TIGIT and the costimulatory molecule ICOS (**Figure 2**).

Like other members of the CD28 family, CTLA-4 can bind CD80/86, but it does so with higher affinity than the co-stimulatory molecule CD28. In the presence of CTLA-4, CD80/86 engagement by CD28 on T cells is inhibited. In addition, CTLA-4 can signal into T cells through Src homology region 2-contatining protein tyrosine phosphatase 2 (SHP-2), dephosphorylating TCR and CD28 signaling intermediates and promoting T-cell inactivation (67). However, engagement of CTLA-4 on T cells by its ligands on APCs can also have inhibitory effects on the latter, such as by triggering the downregulation of CD80 and CD86 (68–70), or by upregulating IDO expression by APCs (71).

LAG-3 is another negative regulator of T-cell activation. This molecule is structurally similar to CD4 and binds MHC class II molecules with higher affinity than CD4 (39). Okazaki’s work has recently shown that LAG-3 does not universally bind to all MHC class II molecules, but rather recognizes stable pMHC class II complexes (72). LAG-3 signals intracellularly, transducing inhibitory signals that hinder T-cell activation (72). Inhibitory signals through the LAG-3 intracytoplasmic region are mediated by a *FXXL* motif in the membrane-proximal region and the EX repeat in the C-terminal region (73). In addition, the LAG-3-pMHCII interaction inhibits DC activation (74).

PD-1 is a co-inhibitory receptor that belongs to the Ig superfamily containing ITIM and ITSM motifs and signals after interacting with its ligands PD-L1 or PD-L2. PD-L1 is expressed on leukocytes, non-hematopoietic cells and non-lymphoid tissues, and can be induced in parenchymal cells by inflammatory cytokines (e.g. IFN-γ) or tumorigenic signaling pathways. PD-L1 expression is also found on different tumor types and is associated with an increased number of tumor-infiltrating lymphocytes (TILs) and poor prognosis. PD-L2 is primarily expressed on professional APCs (DCs and monocytes) but can be induced in other immune and non-immune cell types. PD-1 has a higher binding affinity for PD-L2 than for PD-L1, a difference that might be responsible for the differential contributions of these two ligands to immune responses. It has an inhibitory function similar to that of CTLA-4, by recruiting SHP-1 and SHP-2 phosphatases, reducing T-cell activation and inducing Treg differentiation (75). There is also emerging evidence for ‘reverse signaling’ through PD-L into DCs. PD-1 binding to PD-L2 decreases the expression of DC maturation markers, such as CD40, CD80 and CD86, and increases IL-10 production by DCs, resulting in a suppressive DC phenotype (76).

TIGIT is another immune checkpoint inhibitor that interferes with the activation of T and NK cells. It has an extracellular IgV domain and an intracellular ITT domain that recruits SHIP-1 to mediate T-cell inactivation (77). TIGIT competes with the immunoactivator receptor CD226 (DNAM-1) for the same ligands: CD155 (poliovirus receptor, PVR) and CD112 (Nectin-2 or PVRL2), expressed on APCs, T cells and some non-hematopoietic cell types like tumor cells (78). TIGIT binding to its ligands on APCs has an effect on DC cytokine production, inducing IL-10 expression and inhibiting the expression of IL-12, reducing T-cell activation (79).

ICOS is a costimulatory molecule with structural homology to CD28 and CTLA-4 that binds to ICOS-L on DCs, B cells, and macrophages. ICOS-ICOS-L engagement regulates antigen presentation and secretion of regulatory cytokines such as IL-10 by APCs (80–82).

### Metabolic Disruption

TR1-like cells can also inhibit effector T cells *via* metabolic disruption mechanisms, similar to those used by FoxP3+ Tregs. In TR1-like cells, the main proteins involved in this process are the ectoenzymes ectonucleoside triphosphate diphosphohydrolase 1 (CD39) and ecto-5’-nucleotidase (CD73). These enzymes hydrolyze extracellular 5’-adenosine triphosphate (ATP) to adenosine, disrupting the metabolic state of T cells. ATP released during T-cell activation (83) has an effect on T-cell and APC activation (**Figure 2**). First, CD39 degrades ATP and ADP into AMP (84), which is then further degraded to adenosine by CD73 (85). Adenosine can bind to A_2A_ receptors, inhibiting T-cell proliferation and cytokine production of effector T cells (86). Binding of adenosine to these receptors on APCs inhibits their maturation and the secretion of pro-inflammatory cytokines, while inducing the secretion of IL-10 (87).

### Killing

Another mechanism *via* which TR1-like cells can inhibit T-cell responses is by killing APCs, particularly APCs of myeloid origin. TR1-like cells can express both granzyme A and B proteins, which, together with perforin, mediate cell-mediated cytotoxicity (88) (**Figure 2**). In humans, granzyme expression has been shown to be induced by IL-10 signaling (89). Unlike NK-mediated killing, which takes place when target cells lack or downregulate MHC class I, TR1-mediated cytolysis is antigen-dependent and only takes place when there is TCR engagement with cognate pMHC on the APC (it also requires recognition of other surface molecules expressed by the APC, including CD54 (ICAM-1), CD58, CD155 and CD112) (41). In addition to direct effects on the activation of antigen-specific CD4+ T-cell responses, APC killing indirectly impairs the activation of bystander T cells. Although pMHCII-NP-induced TR1-like cells can express granzymes, they lack cytolytic activity against peptide-pulsed B-cells or DC cells (11).

## Drivers of TR1-Like Cell Formation

### TCR Signaling

TCR stimulation is essential, but not sufficient for the generation of TR1-like cells. pMHC multimers (90–92) or superantigens (93, 94) have been found to induce IL-10-production in some T-cell populations, although it is not clear whether the resulting cells were *bona fide* TR1-like cells. Several studies have suggested that the strength of the TCR interaction plays an important role; high avidity interactions favor IL-10 production (95), in particular the number of IL-10-producing cells and the cells’ suppressive properties (96). The dose of antigen appears to play a lesser role, as high doses of ligands were not enough to induce IL-10 unless they were administered simultaneously with IL-12 (97, 98). Nevertheless, repeated high-dose stimulation was indeed sufficient to induce IL-10. One study pointed to Nfil3 as a transcription factor involved in the upregulation of IL-10 production in response to repeated antigenic stimulation (99). However, as noted above, it is unclear whether these cells were true TR1-like cells or simply Th1 cells that have acquired the ability to produce IL-10. Singha et al. have shown that the ability of pMHCII-NP to elicit TR1 cell formation is dependent on high pMHCII densities onto the NPs. High densities promote sustained pMHC-NP-TCR interactions and formation of TCR microclusters, amplifying the duration and magnitude of TCR signaling, which is associated with their pro-TR1-like cell-differentiation properties (9).

### Interleukin 10

IL-10 has been associated with the induction and maintenance of TR1-like cells (14, 100), although some studies have suggested that it is dispensable (101). In the absence of IL-10 (in *Il10* gene knockout mice) pMHCII-based nanomedicines could readily trigger the expansion of cognate T cells, but these cells upregulated IL-4, suggesting a role for IL-10 in the acquisition of the full-fledged TR1-like cell phenotype (11). Tolerogenic DCs are the main source of IL-10 *in vivo* and they may play a role in the induction of TR1-like cells under physiological conditions (102, 103). Indeed, human IL-10-producing DCs have the ability to induce TR1-like cell differentiation *in vitro* in an IL-10-dependent manner (63, 104).

### Interleukin 27

IL-27, largely produced by activated APCs (105), can support the generation of IL-10-producing TR1-like cells and CD8+ T cells (106, 107). It is a member of the IL-12 family and is a heterodimer composed by the Epstein-Barr virus-induced gene 3 (Ebi3)-encoded IL-12-related p40 and the IL-27 p28 (or IL-27α) chains. IL-27 binds to the IL-27 receptor (IL-27R) on DCs, monocytes, macrophages, T and B lymphocytes, NK cells, mast cells, and endothelial cells. This receptor is a heterodimer composed by the orphan cytokine receptor WSX-1 (also known as T-cell cytokine receptor (TCCR)) and a signal-transducing chain, the glycoprotein 130 (gp130).

**Figure 3:**
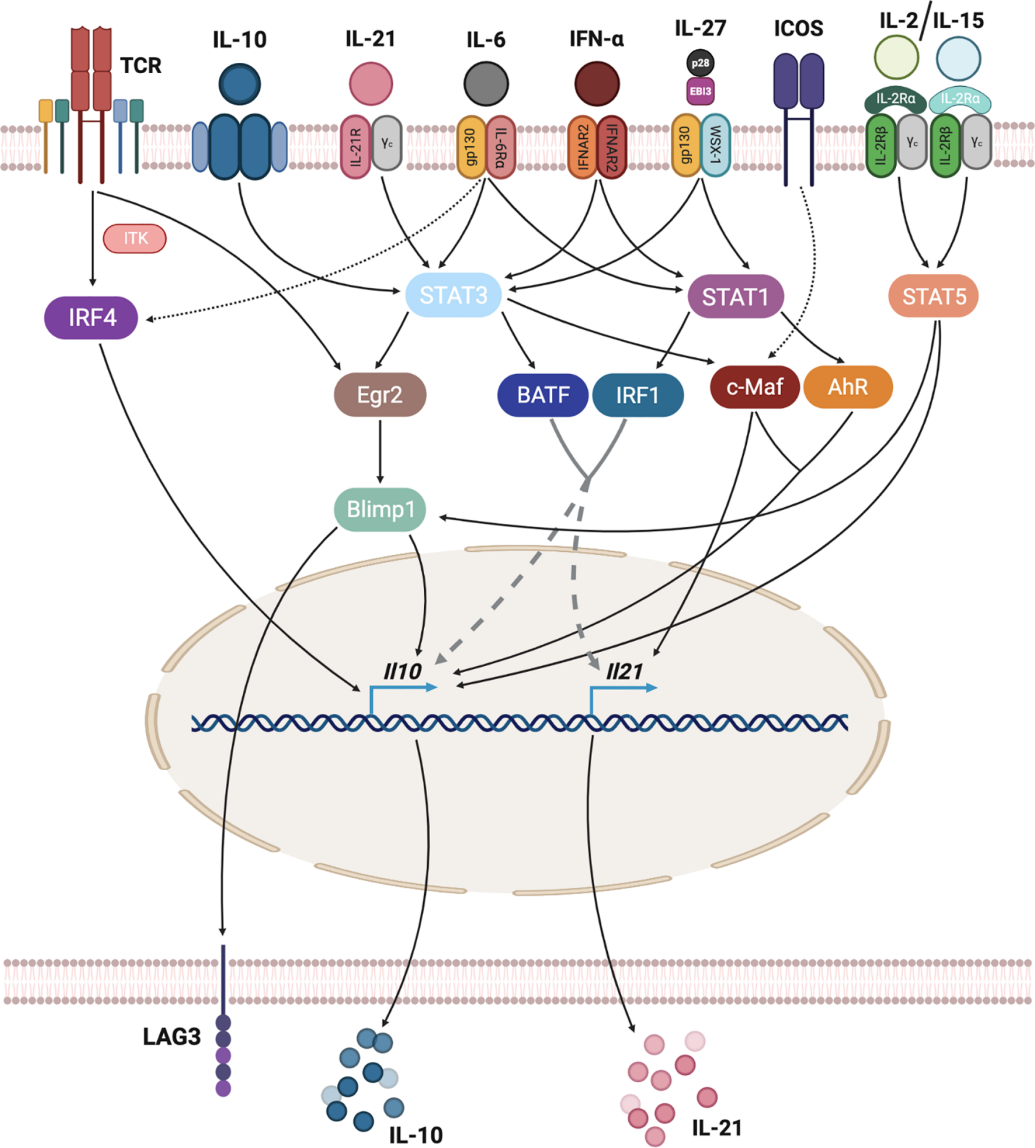
Transcriptional regulation of TR1-like cell formation. TR1-like cell differentiation requires the integration of different stimuli. TCR signaling, through IRF4, can activate IL-10 expression. Many cytokines, including IL-10, IL-21, IL-6, type-I interferons and IL-27 signal *via* STAT1 and/or STAT3 proteins, activating several transcription factors that regulate IL-10 and IL-21 expression. ICOS signaling is also a direct regulator of IL-21 expression, while IL-2 or IL-15 cytokines can induce IL-10 directly *via* STAT5 binding to *Il10* or *via* STAT5-mediated activation of Blimp-1.

IL-27 has inhibitory effects on Th1, Th2 and Th17 subsets as well as on APCs (108–110). Several studies have shown that it is capable of inducing both murine (111, 112) and human (106, 113) IL-10-producing T cells. Signaling through the IL-27R primarily induces STAT1 and STAT3 activation, promoting the expression of AhR and c-Maf transcription factors, which in turn control IL-10 and IL-21 production, hallmarks of the TR1-like cell phenotype (44). STAT3 further upregulates Egr-2, which as noted above contributes to IL-10 production by promoting Blimp-1 expression (57) (**Figure 3**).

Notwithstanding the positive role of IL-27-IL-27R signaling in TR1-like cell differentiation *in vitro*, pMHCII-NP-induced TR1-like cell formation *in vivo* is IL-27R-independent (11).

### Interleukin 21

IL-21 is a type I cytokine that is produced by antigen-stimulated CD4+ T cells as well as NKT cells, and it has pleiotropic effects targeting T, B, NK, and myeloid cells (114). IL-21 binds to a heterodimeric receptor that is composed by the IL-21Rα chain (115) and the common cytokine receptor γ_c_ chain and signals through STAT3 and, to a lesser extent, STAT1 and STAT5. IL-21 plays a critical role in the regulation of Ig production and in the differentiation of B-cells into antibody-producing plasma cells (116, 117), in part by inducing T-follicular helper (TFH) cells (118), and has been implicated in the promotion of CD8+ T-cell and NK cell responses (119). IL-21 can also have negative effects on immune responses, such as by inducing B-cell apoptosis (120) and inhibiting DC maturation and function (121). c-Maf, expressed by TR1-like cells, contributes to IL-21 expression (59), and IL-27 promotes IL-21 expression in TR1-like cells by upregulating c-Maf (44). Furthermore, IL-21 functions as an autocrine growth factor that facilitates the expansion and homeostasis of IL-27-derived TR1-like cells (44), in part by promoting the upregulation of IL-10 (122) and, in turn, c-Maf expression. Like their IL-27-induced counterparts, pMHCII-NP-induced TR1-like cells express and secrete high levels of IL-21 upon recognition of cognate pMHCII on professional APCs (11, 123), which then plays a critical role in the TR1-like cell-induced differentiation of conventional B-cells into IL-10/IL-35-producing Breg cells and in the recruitment/re-programming of neutrophils into myeloid-derived suppressor-like cells, as downstream effectors of pMHCII-NP-induced immunoregulation (11, 123).

### Inducible Costimulator

The ICOS molecule, a member of the B7 superfamily, is a glycosylated disulfide-linked homodimer that is expressed by certain T-cell subsets, including TFH- and TR1-like cells, upon productive TCR ligation. The ICOS-L is expressed on a wide range of lymphoid and non-lymphoid cells types, including APCs (124). ICOS signaling has been implicated in IL-10 production (80), as well as in IL-6-induced TFH cell specification (125, 126), although it can also stimulate the production of Th1 and Th2 cytokines *in vivo*. There is also evidence that c-Maf is a downstream target of ICOS engagement (59, 127), suggesting that ICOS engagement on TR1-like cells plays a role in the stabilization of the TR1-like cell phenotype, in part by sustaining IL-21 and IL-10 expression.

### Interleukin 6

IL-6 is a pleotropic cytokine with both pro- and anti-inflammatory effects. It has been associated with the development/progression of certain autoimmune diseases, such as EAE, rheumatoid arthritis and psoriasis (128–130), in part by promoting Th17, TFH and B-cell responses (131). However, it has suppressive effects on the development of T1D, dextran sodium sulfate (DSS)-induced colitis and inflammatory bone destruction (132–134). IL-6, together with TGF-β, was found to induce expression of IL-10 in Th17 cells without suppressing IL-17 production (112, 135). It has also been shown that IL-6 can upregulate IL-21 production and, together with IL-2, can induce IL-10 expression and thus promote TR1-like cell generation (136), even in the absence of IL-27 or TGF-β. It is worth noting that IL-6 shares certain structural homology with IL-27 and that, like IL-27, binds to the gp130 receptor. Both cytokines signal through STAT1 and STAT3. IL-6 can upregulate the TR1-like transcription factors c-Maf, AhR and IRF4 which, as noted above, play a role in IL-10 and IL-21 production (136).

### Type I Interferons

The type I interferons IFN-α and -β, constitute the first barrier against viral infections by inducing an ‘antiviral state’ in target cells which seeks to blunt protein synthesis, degrade mRNA and promote cell death in order to prevent viral replication. Type I interferons also induce upregulation of MHC I and adhesion molecules to enhance cytotoxic T lymphocyte (CTL)-mediated killing of virus-infected cells. However, IFN-α also has anti-inflammatory properties, such as the suppression of IL-8 and IL-1 production or the upregulation of the IL-1R antagonist (IL-1RA). By signaling *via* STAT1, STAT2 and STAT3 (137, 138), type I IFNs can promote the expression of IL-10 by CD4+ T cells (139–142), including memory T cells. When administered with anti-CD3 and IL-10, IFN-α promoted the development of TR1-like cells (100).

### Interleukin 2 and Interleukin 15

IL-2 and IL-15 function as T-cell growth factors (143, 144). IL-15 was initially shown to play a critical role in the preservation of the memory repertoire, by preventing T-cell apoptosis (145) and promoting the survival of resting memory T cells (146, 147). Some years later, Bacchetta et al. described IL-15 as a growth factor capable of inducing and supporting TR1-like cell proliferation in the absence of TCR ligation (148). Culture of TR1-like cell clones with IL-15 supported their *in vitro* proliferation. A recent report has suggested that IL-15 may inhibit the production of IL-10 by DCs, thus preventing the generation of IL-10-producing CD4+ T cells (149).

The IL-15R shares its β and γ chains with the IL-2R (150, 151). Although similar, IL-2 and IL-15 have non-overlapping functions. While IL-2 is mainly produced by T cells and plays a major role in the homeostasis of IL-2Rα (CD25)-expressing T cells, like activated T cells or nTregs (143, 152), IL-15 is produced during the innate immune response by cell types other than T cells (151). Stimulation with IL-2 can reverse clonal anergy (153). IL-2 and other γ-chain cytokines, such as IL-15 or IL-21, signal through STAT5. The presence of a STAT5-responsive intronic enhancer in the *Il10* locus suggests that these cytokines might also contribute to IL-10 expression by CD4+ T cells (154, 155).

### Role of Antigenic Experience and TR1-Relevant Cytokines in pMHCII-NP-Induced TR1 Cell Formation

The pMHCII-NP-induced TR1 population specifically develops from autoantigen-experienced precursors (11). For example, whereas diabetic NOD.*G6pc2* ^−/−^ mice (which lack IGRP) responded to BDC2.5mi/IA^g7^-NPs like wild-type NOD mice, they did not respond to IGRP_4–22_/IA^g7^-NPs. *In vitro*, BDC2.5 TCR-transgenic anti-CD3/anti-CD28 mAb-activated but not naïve T cells upregulate both CD49b, LAG-3 and IL-10 in response to BDC2.5mi/IA^g7^-NPs, indicating that ligation of cognate TCRs by NP-bound pMHCII complexes can trigger these events only in antigen-experienced cells.

Studies using diabetic NOD.*Ifng^−/−^* and NOD.*Il10* ^−/−^ mice revealed that development of the TR1 precursors and/or TR1-like cells that expand in response to this therapy requires IFN-γ in addition to IL-10 (11). The memory-like phenotype and the upregulation of T-bet mRNA in the expanded TR1-like cells, coupled with the inability of pMHC-NPs to trigger expansion of cognate TR1-like cells in non-diseased mice or NOD.*Ifng^−/−^* mice suggested that the TR1 precursors might be autoantigen-experienced effector/memory T cells of an as yet unknown identity.

As noted above, although IL-27 plays a role in the induction of TR1-like cells from naive T-cell precursors, where it triggers expression of the transcription factor c-Maf, IL-21 and ICOS (44), IL-27 is dispensable for pMHCII-NP-induction of TR1-like cells (11). Since, unlike IL-27, pMHC class II-NPs can only trigger TR1-like cell formation from antigen-experienced but not naive T cells (11), these observations are compatible with the possibility that pMHCII-NPs operate downstream of IL-27.

The specific roles of ICOS, IL-2, IL-6, IL-15 and type I IFNs in the development of pMHCII-NP-induced TR1-like cells remains to be determined.

## Transcriptional Regulation

Transcription factors translate different TR1-like cell-promoting stimuli into transcriptional regulation of key TR1-like cell genes, thus playing a critical role in TR1-like cell specification (**Figure 3**).

### IRF4

The IL-2 inducible T-cell kinase (ITK) plays an essential role in T-cell activation, differentiation and function in response to TCR ligation (156). Although the lack of ITK impairs the development of IL-27-induced TR1-like cells, constitutive expression of the IRF4 (a downstream target of ITK signaling) overcomes this effect (60). Of note, IRF4 expression has been linked to the expression of IL-4 and IL-10 in Th2 cells (157), IL-21, Blimp-1 and Bcl-6 in TFH cells (158) and IL-10 expression in Treg cells (159) or Th1 cells (157).

### c-Maf and AhR

c-Maf has context-dependent effects on IL-4, IL-10 and IL-21 expression. c-Maf positively regulates IL-4 production in both TFH and Th2 cells (160, 161), induces IL-21 expression in both TFH and Th17 cells (59) and contributes to the expression of CXCR5 (162). c-Maf is expressed early on during IL-27-induced TR1-like cell induction, and its expression progressively increases with time (44). IL-27 stimulation also upregulates the expression of AhR, implicated in FoxP3+ Treg and Th17 differentiation (44). c-Maf and AhR have been shown to transactivate both *Il10* and *Il21* gene expression in TR1-like cells (45).

### Egr-2 and Blimp-1

Egr-2 is a transcription factor that plays a role in T-cell anergy (163) and has been associated with the acquisition of regulatory activity by CD4+ T cells (52). Egr-2 expression can be induced by TCR ligation in the absence of costimulation, as well as by IL-27 stimulation (via STAT3). In turn, Egr-2 promotes IL-10 and LAG-3 expression *via* Blimp-1 (57).

The Blimp-1 protein, encoded by the *Prdm1* gene, is a zinc finger-containing transcriptional regulator of plasma cell differentiation (164), but has also been implicated in IL-10 production by CD4+ T cells (165), including both TR1-like (25, 57, 166) and FoxP3+ Treg cells (159).

### IRF1 and BATF

Whereas IL-27R signaling promotes TR1-like cell formation, in part *via* the transcription factors c-Maf, AhR, Egr-2 and Blimp-1, access of these transcription factors to their binding sites on target genes, such as *Il10* or *Il21*, is enabled by pioneering transcription factors, such as BATF and IRF1 (167). BATF had been previously defined as a pioneer factor for Th2, Th17 and effector CD8+ T-cell differentiation, by modifying the chromatin landscape of precursor cells (168–172). BATF also plays a role in TFH differentiation, by regulating Bcl-6 and c-Maf expression (173).

### Other Transcription Factors

Other transcription factors, such as Eomes (174, 175) and Rora (176), have also been proposed to transactivate the *Il10* gene in CD4+ T cells in a context-dependent manner. For example, Eomes requires co-expression of T-bet, the key Th1 transcription factor.

**Figure 3** summarizes the main stimuli leading to TR1-like cell induction, integrating transcriptional regulation of the key TR1-associated genes, *Il10* and *Il21*.

Although pMHCII-NP-induced, antigen-specific TR1-like cells can persist for several months post-treatment withdrawal without any obvious loss of key phenotypic properties or acquisition of pathogenic activity, the cues responsible for their homeostatic survival remain unclear. Cytokines produced by the TR1-like cells themselves or by downstream regulatory cell types (e.g. Breg cells), including IL-10, IL-21 and IL-35, may play a role. Studies employing cell-specific cytokine receptor knock-out mice should help address this knowledge gap.

## IL-10 Upregulation *Versus* TR1-ness

To date, the TR1-ness of specific T-cell types has generally been ascribed to IL-10 expression. However, IL-10 can be expressed by differentiated Th subsets without the need to invoke a true TR1/regulatory phenotype.

For instance, whereas the *Il10* locus lies in a closed conformation in naive CD4+ T cells (177), all differentiated T-helper subsets expose accessible regions along the locus (178), together with deposition of H3K4me3 in the absence of H3K27me3 marks (171, 179), promoting a transcriptionally-competent state. The chromatin remodeling processes that lead to a poised or active *Il10* transcription state in Th subsets is mediated by pioneering transcription factors.

In TR1-like cells, BATF and IRF1 are thought to function as pioneering factors responsible for eliciting some of the chromatin accessibility changes that are required for TR1-like cell differentiation. Only after certain loci, such as *Il10*, become accessible, other TR1-like cell-associated transcription factors, such as AhR and c-Maf, can then bind the *Il10* promoter (167). In Th17 cells, BATF, in association with IRF4, induces *Il10* transcription (180). IRF4 is also involved in eliciting *Il10* expression in Th2 (157, 181) and Treg cells (159). STAT proteins also contribute to *Il10* expression in various Th cell subsets, such as by priming the locus with H3K4me1. STAT4, and STAT6 and GATA-3, induce IL-10 production in Th1 and Th2 cells, respectively (98, 182). GATA-3 induces H3 and H4 acetylation and an increase in chromatin accessibility in the *Il10* locus (183). In Tregs, FoxP3 regulates IL-10 expression, but this process is independent of DNA binding (184). Rather, FoxP3 recruits HAT1 complexes to the locus where they induce the acetylation of H4K5 and H5K12 at the *Il10* promoter, making it more permissive for STAT3 binding (185). Nfil3 is another transcription factor linked to IL-10 production in Th1, Th2, Treg and NK cells, by promoting acetylation of H3 in the *Il10* locus (99). In contrast, in both Th1 and Th2 cells, Ets1 suppresses IL-10 production, by recruiting the de-acetylase HDAC1 to the *Il10* promoter and enhancer regions (186, 187). Importantly, transcription factors involved in T-helper subset specification, such as T-bet, GATA-3 or RORγt can enhance IL-10 expression.

Thus, IL-10 expression *per se* is not a cell subset- but rather a functional state-defining property and IL-10 expression can co-exist with an effector cell program within a given T-cell subset.

## A Role for Epigenetic Remodeling of the Chromatin in TR1-Like Cell Formation?

Transcriptional features alone do not invariably define a final differentiated cell state. Like in other T-cell developmental or differentiation steps, cell fate decisions require both transcriptional changes and epigenetic remodeling of the chromatin.

For example, during development, the epigenome of the parental gametes progressively evolves to acquire the specific epigenome of the zygote (188). The chromatin of the zygote further undergoes additional waves of epigenetic changes, including DNA demethylation and methylation, modifications in histones, and changes in chromatin accessibility (189, 190). Epigenetic reprogramming is also essential for differentiation of embryonic stem cells (ESCs) into distinct cell populations (190–192). Pluripotent cells display an open chromatin configuration that is progressively restricted during development (193), accompanied by an increase in DNA methylation and the redistribution of histone marks. The gene expression changes that are associated with such chromatin remodeling processes are not unique to the germline and also take place in somatic cells in response to stimuli. For example, cytokine stimulation induces chromatin changes in APCs, such as DCs or macrophages (194). This phenomenon has also been reported for cytokine-challenged pancreatic β-cells (195), where cytokine stimulation triggers the appearance of new regulatory elements (neo-IREs).

It is becoming increasingly clear that susceptibility of the chromatin to undergo certain epigenetic modifications is affected by the underlying nucleotide sequence. A significant number of disease-associated single nucleotide polymorphisms (SNPs) lie in fact in non-coding, regulatory regions (196). For example, T1D-associated variants appear to be enriched in T- and B-cell enhancers (196, 197), in some cases promoting a three dimensional chromatin architecture that facilitates changes in gene expression in immune cells that might be able to promote the autoimmune pathology (198). Type 2 diabetes (T2D) is another example of a disease whose genetic susceptibility is commonly associated with non-coding variants (199, 200). In this case, many risk variants locate in enhancers or super-enhancers of genes involved in islet cell function and differentiation (201–204).

**Figure 4:**
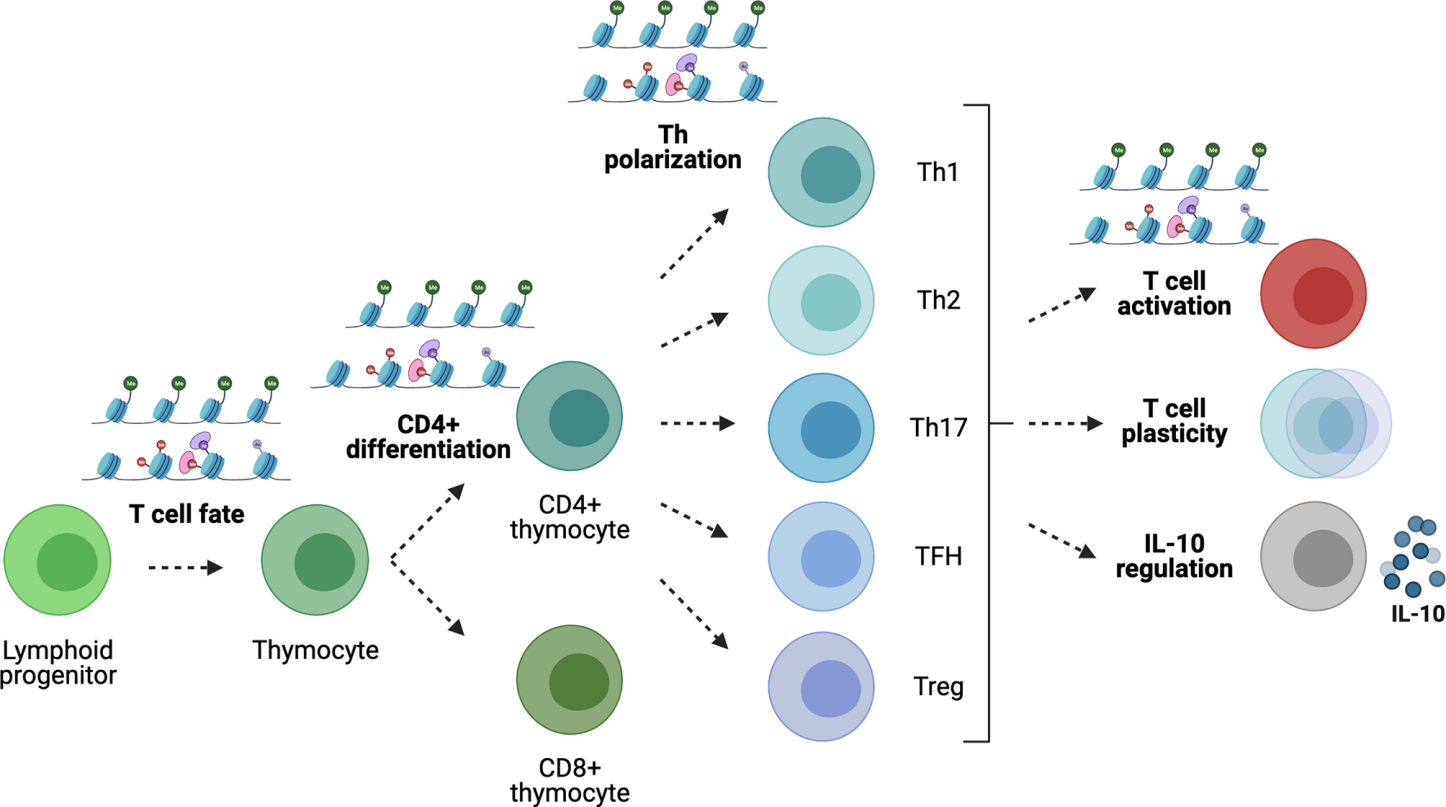
Gene regulation in T cells. Epigenetic modifications play key roles during T-cell development, differentiation and Th polarization. In the periphery, the epigenome regulates Th cell lineage stability/plasticity as well as IL-10 expression competency.

T cells are known to undergo extensive epigenome remodeling in response to activation/differentiation cues, enabling the acquisition of phenotypic and functional stability (**Figure 4**). The first epigenetic decision takes place when the T-cell fate is defined in developing thymocytes (205). T-cell activation (206) and T-helper cell polarization also involve epigenetic modifications along with changes in transcription factor expression. For example, Th1 development involves the upregulation of STAT1 in response to IFN-γ and IL-27, leading to the expression of T-bet, which upregulates the expression of IFN-γ, H2.0-like homeobox (HLX) transcription factors and Runt-related transcription factor 3 (Runx3), and suppresses the expression of GATA-3 (207–209). In turn, T-bet and Runx3 repress the *Il4* gene to prevent Th2 differentiation. The *Ifng* gene harbors multiple regulatory elements around the locus, including enhancers at conserved non-coding sequences and an insulator. This locus is found in a poised, de-methylated state marked by bivalent histone modifications (poised for either expression or silencing) in naive CD4+ T cells, which produce low levels of this cytokine. Th1 differentiation involves H3K4me2, H3 and H4 acetylation and the creation of accessible chromatin at regulatory elements within the *Ifng* locus, together with loss of H3K27me3 throughout the locus, followed by DNA demethylation (210–213). T-bet transactivates expression of *Ifng* by binding to its promoter as well as several enhancers and by recruiting histone acetyltransferases (HATs) (214) and histone demethylases (HDMs) (215).

In contrast, activation of the Th2 program results in the loss of permissive histone modifications and H3K27 trimethylation along the *Ifng* locus, coupled to DNA methylation (210, 216, 217). The Th2 program is induced by IL-4-mediated activation of STAT6, which in turn activates GATA-3 (218). GATA-3 induces the expression of c-Maf, regulating IL-4 expression, and together with STAT6 enhances the transcription of *Il4*, *Il5* and *Il13* (218). In mice, *Il4*, *Il5* and *Il13* (encoding Th2 cytokines), together with the *Rad50* gene, co-localize near a Locus Control Region (LCR). Expression of the *Il4* gene is regulated by enhancers (overlapping with DNAse I hypersensitive sites) that bind NFAT and Th2-promoting transcription factors. In naive T cells, there are few accessibility and histone modifications at these DNAse I hypersensitive sites, and the cytokine gene promoters and enhancers are hyper-methylated (219). Upon Th2-polarizing stimulation, the loci acquire permissive histone modifications and lose H3K27me3 (220, 221). In Th1 cells, the Th2-cytokine locus is all covered with H3K27me3 (222). GATA-3 induces most of these epigenetic modifications, as it can recruit HATs and histone H3K4 methyltransferases (218, 223), inhibit histone deacetylases (HDACs) (224) and DNA (cytosine-5)-methyltransferase 1 (DNMT1) (219, 225), and recruit chromatin-remodeling factors (226). In addition, the *Ifng* locus in Th2 cells is silenced by H3K27me3 deposition (217).

TGF-β induction of Th17 and Treg lineage formation represents another example. This cytokine induces both the expression of FoxP3 and retinoic-acid-receptor-related orphan receptor-γt (RORγt) (227–230). The context determines if the Treg or the Th17 program is induced: in the absence of IL-6, FoxP3 inhibits RORγt and leads to Treg formation. If IL-6 is present, STAT3 is activated, inhibiting the expression of FoxP3 and enhancing Th17 formation. IL-17A and IL-17F are both co-expressed by Th17 cells and the genes encoding these cytokines co-localize and may be regulated by shared regulatory elements. The *Il17* locus contains eight different gene regulatory elements (231). When naïve CD4+ T cells are cultured under Th17-polarizing conditions, STAT3 (227) and RORγt (232) induce the appearance of permissive H3 acetylation changes in the *Il17a* and *Il17f* gene regulatory elements (231), enabling their expression.

The fate of TFH and non-TFH (Th1, Th2, Th17) effector cells is regulated by Bcl-6 and Blimp-1, which are reciprocal regulators of each other (233). Bcl-6 binds promoters and enhancers regulating genes involved in T-cell migration (Ebi2, CCR6, CCR7, S1pr1, Klf-2, PSGL-1, CXCR5, CXCR4, PD-1 and SAP) (126, 234). Ascl-2 also controls TFH differentiation by upregulating CXCR5 and CXCR4, while downregulating CCR7 and PSGL-1 expression. The *Bcl6* locus in TFH cells displays positive histone modifications, but it also contains permissive marks in Th1, Th2 and Th17 cells (235). Other TFH-related genes, such as c-Maf, BATF and IRF4, are also associated with H3K4me3 in all subsets. In contrast, the *Ascl2* locus is uniquely marked with the active chromatin mark H3K4me3 in TFH cells. *Prdm1* (encoding BLIMP-1), which is downregulated in TFH cells, displays bivalent modifications, allowing re-programming between TFH and other Th effector subsets (236).

Acquisition of Treg-cell-specific epigenetic marks during thymocyte development (237, 238), along with FoxP3 expression, determines the regulatory phenotype of nTregs (239). Once in the periphery, Treg cells can be divided into subpopulations that locate in different tissues, and each acquires an additional level of epigenetic modification that defines a tissue-specific epigenetic footprint (240). The expression of some Treg-function associated molecules, such as CTLA-4 or CD25, is clearly associated with DNA de-methylation and can occur in the absence of FoxP3. In contrast, expression of *Il2*, *Ifng* or *Zap70* is lost if FoxP3 is not present. Several regulatory elements control *Foxp3* gene expression (237). The *Foxp3* promoter is de-methylated upon TCR signaling, facilitating the binding of FoxP3-inducing transcription factors (241).

Moreover, there is increasing evidence that Th subsets are plastic. To name a few examples, T-cell populations have been described that stably express both T-bet and GATA-3 and produce both IFN-γ and IL-4 (242), produce both Th1 and Th17 cytokines (243, 244), or have a Th1/Th17 phenotype but can switch to Th2 during helminth infections (245). This observed plasticity can be regulated by different mechanisms. First, certain environmental stimuli may be able to modify epigenetic marks responsible for maintaining lineage stability; for example, prolonged activation of Treg cells *in vitro* can lead to demethylation of the *Rorc* locus in FoxP3+ Tregs and allow IL-17 production (246). Second, Th subsets display an intrinsic plasticity potential. Although the various lineage-specific cytokines present active histone marks in the corresponding cell lineages and repressive marks in the others, some transcription factors are not so strictly marked. For example, in Th1 cells, *Tbx21*, encoding T-bet, bears activating H3K4me3 marks in the promoter. In other Th cell subsets, on the other hand, the *Tbx21* promoter bears bivalent modifications. Likewise, the *Gata3* promoter carries H3K4me3 marks in Th2 cells, but bivalent marks in other Th cell subsets. The same is true for *Rorc* or *Bcl6* genes in non-Th17 or non-TFH cells, respectively (235, 247). Third, polarized T-cell types might represent stable lineages yet comprise a continuum of different epigenotypes with differential susceptibility for lineage conversion in response to external signals.

To date, the study of TR1-like cell specification is largely based on phenotypic and transcriptional studies. Based on the data summarized above, it is reasonable to suspect that the cues responsible for TR1-like cell formation operate on a precursor cell type that either has a TR1-poised epigenome or responds to TR1-inducing signals by undergoing further epigenetic modifications enabling the acquisition of a stable TR1-like cell phenotype, including DNA hypomethylation (248–250). Thus, detailed characterization of TR1-like cells at the transcriptional and epigenetic levels, including analysis of their chromatin status, three-dimensional structure and interactions, as well as DNA methylation status (**Table 2**), should provide unique clues about the true identity of this cell lineage and the identity of their cellular precursors.

**Table 2:** Summary of epigenetic modifications and their effects on gene expression.

*Epigenetic modification*	*Found in*	*Relation to gene transcription*	*Study methods*
*Chromatin accessibility*	Promoters and GREs	Activating	ATAC-seq DNAse-seq MNase-seq
*H3K27ac*	Active enhancers and TSS	Activating	
*H3K4me3*	TSS	Activating	
*H3K4me1*	Gene body Primed enhancers	Activating	ChIP-seq Cut&Run
*H3K27me3*	Bivalent/inactive enhancers, promoters and intergenic regions	Repressing	Cut&Tag
*H3K9me3*	Constitutive heterochromatin	Repressing	
*DNA methylation*	Enhancers, promoters, gene body (CpG rich regions)	Repressing	Bisulfite sequencing

## Concluding Remarks

Currently, TR1-like cells are defined as a regulatory CD4+ T-cell subset that lacks FoxP3 expression (unlike conventional FoxP3+ Treg cells) and secretes IL-10 and low levels or no IL-4. However, this Treg cell subset lacks cell-specific markers and their developmental origin remains a mystery. Moreover, the signaling, genetic and epigenetic mechanisms that are responsible for the acquisition of the TR1-like cell phenotype *in vivo* remain unclear.

We posit that detailed transcriptional and epigenetic studies will enable a better understanding of the role of this T-cell subset in immunity, autoimmunity and cancer, the identification of biomarkers capable of accurately track its development *in vivo*, as well as a detailed understanding of gene regulatory mechanisms responsible for TR1-like cell specification. In turn, this will help pinpoint specific areas of the genome that might be impacted by genetic polymorphisms associated with susceptibility and/or resistance to specific immune-mediated diseases, and the development and testing of compounds capable of triggering the formation of antigen-specific TR1-like cells *in vivo*, for therapeutic purposes.

TR1 cell formation in response to pMHCII-NPs afford a unique opportunity to address the above knowledge gaps. These compounds trigger the formation of relatively large numbers of mono-specific TR1-like cells, thus enabling this type of studies with unprecedented resolution. Transcriptional studies at the bulk and single cell levels should help determine the homogeneity or heterogeneity of the resulting cognate T-cell pools, and pinpoint developmentally-related cell subsets with poised TR1-like cell transcriptional programs. Epigenetic studies shall include screening for histone modifications, chromatin accessibility and 3D chromatin maps, to enumerate the genome-wide distribution of active promoters and enhancers, define the epigenomic architecture underpinning the TR1-like cell state, describe the various steps underlying TR1-like cell re-programming, identify key TR1-like cell epigenetic signatures, and potentially expose new targets for therapeutic intervention.

Collectively, these studies should provide a comprehensive set of functional, phenotypic, transcriptional and epigenetic markers capable of specifically identifying TR1 cells. These markers would likely play a pivotal role in guiding the clinical translation of compounds capable of promoting TR1 cell formation *in vivo* for the treatment of autoimmunity, including pMHCII-based nanomedicines. They should also prove useful to enumerate the contribution of this cell type to tumor progression in the context of cancer.

## Author Contributions

PSo and PSa co-wrote the manuscript. All authors contributed to the article and approved the submitted version.

## Funding

The authors’ work is funded by the Canadian Institutes of Health Research (CIHR), Diabetes Canada, REEM (Red Española de Esclerosis Múltiple), the Praespero Foundation, the Ministerio de Economia y Competitividad of Spain (MINECO; RD16/0015/0020), and Generalitat de Catalunya (SGR and CERCA Programmes). The JMDRC is supported by Diabetes Canada.

## Conflict of Interest

PSa is scientific founder of Parvus Therapeutics Inc. and has a financial interest in the company.

The remaining author declares that the research was conducted in the absence of any commercial or financial relationships that could be construed as a potential conflict of interest.

## References

[1] Fujio K Okamura T Yamamoto K. The Family of IL-10-Secreting CD4+T Cells. In: Advances in Immunology. Elsevier Inc. (2010). p. 99–130. 10.1016/S0065-2776(10)05004-2 20510731

[2] Alfen JS Larghi P Facciotti F Gagliani N Bosotti R Paroni M. Intestinal IFN-γ–Producing Type 1 Regulatory T Cells Coexpress CCR5 and Programmed Cell Death Protein 1 and Downregulate IL-10 in the Inflamed Guts of Patients With Inflammatory Bowel Disease. J Allergy Clin Immunol (2018) 142:1537–47.e8. 10.1016/j.jaci.2017.12.984 29369775

[3] Gagliani N Magnani CF Huber S Gianolini ME Pala M Licona-Limon P. Coexpression of CD49b and LAG-3 Identifies Human and Mouse T Regulatory Type 1 Cells. Nat Med (2013) 19:739–46. 10.1038/nm.3179 23624599

[4] Roncarolo MG Gregori S Bacchetta R Battaglia M Gagliani N. The Biology of T Regulatory Type 1 Cells and Their Therapeutic Application in Immune-Mediated Disease. Immunity (2018) 49:1004–19. 10.1016/j.immuni.2018.12.001 30566879

[5] Burton BR Britton GJ Fang H Verhagen J Smithers B Sabatos-Peyton CA. Sequential Transcriptional Changes Dictate Safe and Effective Antigen-Specific Immunotherapy. Nat Commun (2014) 5:4741. 10.1038/ncomms5741 25182274 PMC4167604

[6] White AM Wraith DC. Tr1-Like T Cells - An Enigmatic Regulatory T Cell Lineage. Front Immunol (2016) 7:355. 10.3389/fimmu.2016.00355 27683580 PMC5021682

[7] Brockmann L Soukou S Steglich B Czarnewski P Zhao L Wende S. Molecular and Functional Heterogeneity of IL-10-Producing CD4+ T Cells. Nat Commun (2018) 9:5457. 10.1038/s41467-018-07581-4 30575716 PMC6303294

[8] Zhu C Sakuishi K Xiao S Sun Z Zaghouani S Gu G. An IL-27/NFIL3 Signaling Axis Drives Tim-3 and IL-10 Expression and T Cell Dysfunction. Nat Commun (2015) 6:1–14. 10.1038/ncomms7072 PMC4311884 25614966

[9] Singha S Shao K Yang Y Clemente-Casares X Solé P Clemente A. Peptide–MHC-Based Nanomedicines for Autoimmunity Function as T-Cell Receptor Microclustering Devices. Nat Nanotechnol (2017) 12:701–10. 10.1038/nnano.2017.56 28436959

[10] Tsai S Shameli A Yamanouchi J Clemente-Casares X Wang J Serra P. Reversal of Autoimmunity by Boosting Memory-Like Autoregulatory T Cell. Immunity (2010) 32:568–80. 10.1016/j.immuni.2010.03.015 20381385

[11] Clemente-Casares X Blanco J Ambalavanan P Yamanouchi J Singha S Fandos C. Expanding Antigen-Specific Regulatory Networks to Treat Autoimmunity. Nature (2016) 530:434–40. 10.1038/nature16962 26886799

[12] Umeshappa CS Singha S Blanco J Shao K Nanjundappa RH Yamanouchi J. Suppression of a Broad Spectrum of Liver Autoimmune Pathologies by Single Peptide-MHC-Based Nanomedicines. Nat Commun (2019) 10:1–17. 10.1038/s41467-019-09893-5 31089130 PMC6517389

[13] Umeshappa CS Mbongue J Singha S Mohapatra S Yamanouchi J Lee JA. Ubiquitous Antigen-Specific T Regulatory Type 1 Cells Variably Suppress Hepatic and Extrahepatic Autoimmunity. J Clin Invest (2020) 130:1823–9. 10.1172/JCI130670 PMC7108901 32125290

[14] Groux H O’Garra A Bigler M Rouleau M Antonenko S de Vries JE. Roncarolo MG. A CD4+ T-Cell Subset Inhibits Antigen-Specific T-Cell Responses and Prevents Colitis. Nature (1997) 389:737–42. 10.1038/39614 9338786

[15] Roncarolo MG Yssel H Touraine JL Betuel H De Vries JE Spits H. Autoreactive T Cell Clones Specific for Class I and Class II HLA Antigens Isolated From a Human Chimera. J Exp Med (1988) 167:1523–34. 10.1084/jem.167.5.1523 PMC2188931 3284961

[16] Vieira P De Waal-Malefyt R Dang MN Johnson KE Kastelein R Fiorentino DF. Isolation and Expression of Human Cytokine Synthesis Inhibitory Factor cDNA Clones: Homology to Epstein-Barr Virus Open Reading Frame BCRF. Proc Natl Acad Sci USA (1991) 88:1172–6. 10.1073/pnas.88.4.1172 PMC50979 1847510

[17] Bacchetta R Bigler M Touraine JL Parkman R Tovo PA Abrams J. High Levels of Interleukin 10 Production *In Vivo* Are Associated With Tolerance in SCID Patients Transplanted With HLA Mismatched Hematopoietic Stem Cells. J Exp Med (1994) 179:493–502. 10.1084/jem.179.2.493 7905018 PMC2191349

[18] Bacchetta R Gregori S Serafini G Sartirana C Schulz U Zino E. Molecular and Functional Characterization of Allogantigen Specific Anergic T Cells Suitable for Cell Therapy. Haematologica (2010) 95:2134–43. 10.3324/haematol.2010.025825 PMC2995573 20713457

[19] Roncarolo MG Gregori S Bacchetta R Battaglia M. Tr1 Cells and the Counter-Regulation of Immunity: Natural Mechanisms and Therapeutic Applications. Curr Top Microbiol Immunol (2014) 380:39–68. 10.1007/978-3-662-43492-5_3 25004813

[20] Bollyky PL Wu RP Falk BA Lord JD Long SA Preisinger A. ECM Components Guide IL-10 Producing Regulatory T-Cell (TR1) Induction From Effector Memory T-Cell Precursors. Proc Natl Acad Sci USA (2011) 108:7938–43. 10.1073/pnas.1017360108 PMC3093524 21518860

[21] Yao Y Vent-Schmidt J McGeough MD Wong M Hoffman HM Steiner TS. Tr1 Cells, But Not Foxp3 + Regulatory T Cells, Suppress NLRP3 Inflammasome Activation *via* an IL-10–Dependent Mechanis. J Immunol (2015) 195:488–97. 10.4049/jimmunol.1403225 26056255

[22] Gagliani N Gregori S Jofra T Valle A Stabilini A Rothstein DM. Rapamycin Combined With Anti-CD45RB mAB and IL-10 or With G-CSF Induces Tolerance in a Stringent Mouse Model of Islet Transplantation. PloS One (2011) 6:1–12. 10.1371/journal.pone.0028434 PMC3235119 22174806

[23] Gagliani N Jofra T Valle A Stabilini A Morsiani C Gregori S. Transplant Tolerance to Pancreatic Islets Is Initiated in the Graft and Sustained in the Spleen. Am J Transplant (2013) 13:1963–75. 10.1111/ajt.12333 PMC3869180 23834659

[24] Gagliani N Amezcua Vesely MC Iseppon A Brockmann L Xu H Palm NW. TH17 Cells Transdifferentiate Into Regulatory T Cells During Resolution of Inflammation. Nature (2015) 523:221–5. 10.1038/nature14452 PMC4498984 25924064

[25] Heinemann C Heink S Petermann F Vasanthakumar A Rothhammer V Doorduijn E. IL-27 and IL-12 Oppose Pro-Inflammatory IL-23 in CD4+ T Cells by Inducing Blimp1. Nat Commun (2014) 5:1–13. 10.1038/ncomms4770 24796719

[26] Meiron M Zohar Y Anunu R Wildbaum G Karin N. CXCL12 (SDF-1α) Suppresses Ongoing Experimental Autoimmune Encephalomyelitis by Selecting Antigen-Specific Regulatory T Cells. J Exp Med (2008) 205:2643–55. 10.1084/jem.20080730 PMC2571938 18852294

[27] Lönnberg T Svensson V James KR Fernandez-Ruiz D Sebina I Montandon R. Single-Cell RNA-Seq and Computational Analysis Using Temporal Mixture Modeling Resolves TH1/TFH Fate Bifurcation in Malaria. Sci Immunol (2017) 2(9):eaal2192. 10.1126/sciimmunol.aal2192 28345074 PMC5365145

[28] Pacciani V Gregori S Chini L Corrente S Chianca M Moschese V. Induction of Anergic Allergen-Specific Suppressor T Cells Using Tolerogenic Dendritic Cells Derived From Children With Allergies to House Dust Mites. J Allergy Clin Immunol (2010) 125:727–36. 10.1016/j.jaci.2009.12.004 20153036

[29] Pellerin L Jenks JA Chinthrajah S Dominguez T Block W Zhou X. Peanut-Specific Type 1 Regulatory T Cells Induced *In Vitro* From Allergic Subjects Are Functionally Impaired. J Allergy Clin Immunol (2018) 141:202–213.e8. 10.1016/j.jaci.2017.05.045 28689791

[30] Oida T Zhang X Goto M Hachimura S Totsuka M Kaminogawa S. CD4+ CD25– T Cells That Express Latency-Associated Peptide on the Surface Suppress CD4+ CD45RB High -Induced Colitis by a TGF-β-Dependent Mechanis. J Immunol (2003) 170:2516–22. 10.4049/jimmunol.170.5.2516 12594277

[31] Ochi H Abraham M Ishikawa H Frenkel D Yang K Basso AS. Oral CD3-Specific Antibody Suppresses Autoimmune Encephalomyelitis by Inducing CD4+CD25–LAP+ T Cells. Nat Med (2006) 12:627–35. 10.1038/nm1408 16715091

[32] Groh V Smythe K Dai Z Spies T. Fas Ligand-Mediated Paracrine T Cell Regulation by the Receptor NKG2D in Tumor Immunity. Nat Immunol (2006) 7:755–62. 10.1038/ni1350 16732291

[33] Groh V Brühl A El-Gabalawy H Nelson JL Spies T. Stimulation of T Cell Autoreactivity by Anomalous Expression of NKG2D and Its MIC Ligands in Rheumatoid Arthritis. Proc Natl Acad Sci USA (2003) 100:9452–7. 10.1073/pnas.1632807100 PMC170939 12878725

[34] Häringer B Lozza L Steckel B Geginat J. Identification and Characterization of IL-10/IFN-γ-Producing Effector-Like T Cells With Regulatory Function in Human Blood. J Exp Med (2009) 206:1009–17. 10.1084/jem.20082238 PMC2715038 19414553

[35] Gonzalez A Andre-Schmutz I Carnaud C Mathis D Benoist C. Damage Control, Rather Than Unresponsiveness, Effected by Protective DX5+T Cells in Autoimmune Diabetes. Nat Immunol (2001) 2:1117–25. 10.1038/ni738 11713466

[36] Charbonnier L-M van Duivenvoorde LM Apparailly F Cantos C Han WGH Noël D. Immature Dendritic Cells Suppress Collagen-Induced Arthritis by In Vivo Expansion of CD49b + Regulatory T Cell. J Immunol (2006) 177:3806–13. 10.4049/jimmunol.177.6.3806 16951342

[37] Han WGH Schuurhuis DH Fu N Camps M van Duivenvoorde LM Louis-Plence P. DC-Induced CD8+ T-Cell Response Is Inhibited by MHC Class II-Dependent DX5+CD4+ Treg. Eur J Immunol (2009) 39:1765–73. 10.1002/eji.200838842 19544486

[38] El Bannoudi H Han WGH Stoop JN Louis-Plence P Huizinga TWJ Toes REM. DX5+CD4+ T Cells Modulate CD4+ T-Cell Response *via* Inhibition of IL-12 Production by DC. Eur J Immunol (2013) 43:439–46. 10.1002/eji.201242796 23169063

[39] Workman CJ Rice DS Dugger KJ Kurschner C Vignali DAA. Phenotypic Analysis of the Murine CD4-Related Glycoprotein, CD223 (LAG-3). Eur J Immunol (2002) 32:2255–63. 10.1002/1521-4141(200208)32:8<2255::AID-IMMU2255>3.0.CO;2-A 12209638

[40] Facciotti F Gagliani N Häringer B Alfen JS Penatti A Maglie S. IL-10-Producing Forkhead Box Protein 3-Negative Regulatory T Cells Inhibit B-Cell Responses and Are Involved in Systemic Lupus Erythematosus. J Allergy Clin Immunol (2016) 137:318–21. 10.1016/j.jaci.2015.06.044 26318071

[41] Magnani CF Alberigo G Bacchetta R Serafini G Andreani M Roncarolo MG. Killing of Myeloid APCs *via* HLA Class I, CD2 and CD226 Defines a Novel Mechanism of Suppression by Human Tr1 Cells. Eur J Immunol (2011) 41:1652–62. 10.1002/eji.201041120 PMC3116154 21469116

[42] Cobbold SP Nolan KF Graca L Castejon R Le Moine A Frewin M. Regulatory T Cells and Dendritic Cells in Transplantation Tolerance: Molecular Markers and Mechanisms. Immunol Rev (2003) 196:109–24. 10.1046/j.1600-065X.2003.00078.x 14617201

[43] Okamura T Fujio K Shibuya M Sumitomo S Shoda H Sakaguchi S. CD4+CD25-LAG3+ Regulatory T Cells Controlled by the Transcription Factor Egr-2. Proc Natl Acad Sci (2009) 106:13974–9. 10.1073/pnas.0906872106 PMC2729005 19666526

[44] Pot C Jin H Awasthi A Liu SM Lai C-Y Madan R. Cutting Edge: IL-27 Induces the Transcription Factor C-Maf, Cytokine IL-21, and the Costimulatory Receptor ICOS That Coordinately Act Together to Promote Differentiation of IL-10-Producing Tr1 Cell. J Immunol (2009) 183:797–801. 10.4049/jimmunol.0901233 19570826 PMC2768608

[45] Apetoh L Quintana FJ Pot C Joller N Xiao S Kumar D. The Aryl Hydrocarbon Receptor Interacts With C-Maf to Promote the Differentiation of Type 1 Regulatory T Cells Induced by IL-27. Nat Immunol (2010) 11:854–61. 10.1038/ni.1912 PMC2940320 20676095

[46] Tousa S Semitekolou M Morianos I Banos A Trochoutsou AI Brodie TM. Activin-A Co-Opts IRF4 and AhR Signaling to Induce Human Regulatory T Cells That Restrain Asthmatic Responses. Proc Natl Acad Sci USA (2017) 114:2891–900. 10.1073/pnas.1616942114 PMC5389328 28320933

[47] Nakamura K Kitani A Strober W. Cell Contact-Dependent Immunosuppression by CD4+CD25+ Regulatory T Cells Is Mediated by Cell Surface-Bound Transforming Growth Factor β. J Exp Med (2001) 194:629–44. 10.1084/jem.194.5.629 PMC2195935 11535631

[48] Nakajima U Miyazono K Kato M Takase M Yamagishi T Nakamura H. Extracellular Fibrillar Structure of Latent Tgfβ Binding Protein-1: Role in Tgfβ-Dependent Endothelial-Mesenchymal Transformation During Endocardial Cushion Tissue Formation in Mouse Embryonic Heart. J Cell Biol (1997) 136:193–204. 10.1083/jcb.136.1.193 9008713 PMC2132455

[49] Bradley LM Haynes L Swain SL. IL-7: Maintaining T-Cell Memory and Achieving Homeostasis. Trends Immunol (2005) 26:172–6. 10.1016/j.it.2005.01.004 15745860

[50] Liu W Putnam AL Xu-yu Z Szot GL Lee MR Zhu S. CD127 Expression Inversely Correlates With FoxP3 and Suppressive Function of Human CD4+ T Reg Cells. J Exp Med (2006) 203:1701–11. 10.1084/jem.20060772 PMC2118339 16818678

[51] Huang C-TT Workman CJ Flies D Pan X Marson AL Zhou G. Role of LAG-3 in Regulatory T Cells. Immunity (2004) 21:503–13. 10.1016/j.immuni.2004.08.010 15485628

[52] Safford M Collins S Lutz MA Allen A Huang CT Kowalski J. Egr-2 and Egr-3 Are Negative Regulators of T Cell Activation. Nat Immunol (2005) 6:472–80. 10.1038/ni1193 15834410

[53] Kassiotis G Gray D Kiafard Z Zwirner J Stockinger B. Functional Specialization of Memory Th Cells Revealed by Expression of Integrin CD49. J Immunol (2006) 177:968–75. 10.4049/jimmunol.177.2.968 16818752

[54] Gupta S Thornley TB Gao W Larocca R Turka LA Kuchroo VK. Allograft Rejection Is Restrained by Short-Lived TIM 3^+^ PD1^+^ Foxp3^+^ Treg. J Clin Invest (2012) 122:2395–404. 10.1172/JCI45138 PMC3386804 22684103

[55] Wu H Chen Y Liu H Xu LL Teuscher P Wang S. Follicular Regulatory T Cells Repress Cytokine Production by Follicular Helper T Cells and Optimize IgG Responses in Mice. Eur J Immunol (2016) 46:1152–61. 10.1002/eji.201546094 PMC4896226 26887860

[56] Miaw SC Choi A Yu E Kishikawa H Ho IC. ROG, Repressor of GATA, Regulates the Expression of Cytokine Genes. Immunity (2000) 12:323–33. 10.1016/s1074-7613(00)80185-5 10755619

[57] Iwasaki Y Fujio K Okamura T Yanai A Sumitomo S Shoda H. Egr-2 Transcription Factor Is Required for Blimp-1-Mediated IL-10 Production in IL-27-Stimulated CD4+T Cells. Eur J Immunol (2013) 43:1063–73. 10.1002/eji.201242942 23349024

[58] Veldhoen M Hirota K Westendorf AM Buer J Dumoutier L Renauld J-C. The Aryl Hydrocarbon Receptor Links TH17-Cell-Mediated Autoimmunity to Environmental Toxins. Nature (2008) 453:106–9. 10.1038/nature06881 18362914

[59] Bauquet AT Jin H Paterson AM Mitsdoerffer M Ho I-CC Sharpe AH. The Costimulatory Molecule ICOS Regulates the Expression of C-Maf and IL-21 in the Development of Follicular T Helper Cells and TH -17 Cells. Nat Immunol (2009) 10:167–75. 10.1038/ni.1690 PMC2742982 19098919

[60] Huang W Solouki S Koylass N Zheng S-GG August A. ITK Signalling *via* the Ras/IRF4 Pathway Regulates the Development and Function of Tr1 Cells. Nat Commun (2017) 8:15871. 10.1038/ncomms15871 28635957 PMC5482062

[61] Moore KW de Waal Malefyt R Coffman RL O’Garra A. Interleukin-10 and the Interleukin-10 Receptor. Annu Rev Immunol (2001) 19:683–765. 10.1146/annurev.immunol.19.1.683 11244051

[62] Taga K Tosato G. IL-10 Inhibits Human T Cell Proliferation and IL-2 Production. J Immunol (1992) 148:1143–8. 1737931

[63] Gregori S Tomasoni D Pacciani V Scirpoli M Battaglia M Magnani CF. Differentiation of Type 1 T Regulatory Cells (Tr1) by Tolerogenic DC-10 Requires the IL-10-Dependent ILT4/HLA-G Pathway. Blood (2010) 116:935–44. 10.1182/blood-2009-07-234872 20448110

[64] Satoguina JS Weyand E Larbi J Hoerauf A. T Regulatory-1 Cells Induce IgG4 Production by B Cells: Role of IL-10. J Immunol (2005) 174:4718–26. 10.4049/jimmunol.174.8.4718 15814696

[65] Murray PJ. The Primary Mechanism of the IL-10-Regulated Antiinflammatory Response Is to Selectively Inhibit Transcription. Proc Natl Acad Sci USA (2005) 102:8686–91. 10.1073/pnas.0500419102 PMC1150817 15937121

[66] Bourque J Hawiger D. Immunomodulatory Bonds of the Partnership Between Dendritic Cells and T Cells. Crit Rev Immunol (2018) 38:379–401. 10.1615/CritRevImmunol.2018026790 30792568 PMC6380512

[67] Lee KM Chuang E Griffin M Khattri R Hong DK Zhang W. Molecular Basis of T Cell Inactivation by CTLA-4. Science (1998) 282:2263–6. 10.1126/science.282.5397.2263 9856951

[68] Serra P Amrani A Yamanouchi J Han B Thiessen S Utsugi T. CD40 Ligation Releases Immature Dendritic Cells From the Control of Regulatory CD4+CD25+ T Cell. Immunity (2003) 19:877–89. 10.1016/S1074-7613(03)00327-3 14670304

[69] Oderup C Cederbom L Makowska A Cilio CM Ivars F. Cytotoxic T Lymphocyte Antigen-4-Dependent Down-Modulation of Costimulatory Molecules on Dendritic Cells in CD4+ CD25+ Regulatory T-Cell-Mediated Suppression. Immunology (2006) 118:240–9. 10.1111/j.1365-2567.2006.02362.x PMC1782280 16771859

[70] Cederbom L Hall H Ivars F. Stimulatory Molecules on Antigen-Presenting Cell. Cell (2000) 30:1538–43. 10.1002/1521-4141(200006)30:6<1538::AID-IMMU1538>3.0.CO;2-X 10898488

[71] Finger EB Bluestone JA. When Ligand Becomes Receptor — Tolerance via B7 Signaling on DC. Nat Immunol (2002) 3:1056–7. 10.1038/ni1102-1056 12407416

[72] Maruhashi T Okazaki II mi Sugiura D Takahashi S Maeda TK Shimizu K. LAG-3 Inhibits the Activation of CD4 + T Cells That Recognize Stable pMHCII Through Its Conformation-Dependent Recognition of pMHCII. Nat Immunol (2018) 19:1415–26. 10.1038/s41590-018-0217-9 30349037

[73] Maeda TK Sugiura D Okazaki II mi Maruhashi T Okazaki T. Atypical Motifs in the Cytoplasmic Region of the Inhibitory Immune Co-Receptor LAG-3 Inhibit T Cell Activation. J Biol Chem (2019) 294:6017–26. 10.1074/jbc.RA119.007455 PMC6463702 30760527

[74] Liang B Workman C Lee J Chew C Dale BM Colonna L. Regulatory T Cells Inhibit Dendritic Cells by Lymphocyte Activation Gene-3 Engagement of MHC Class II. J Immunol (2008) 180:5916–26. 10.4049/jimmunol.180.9.5916 18424711

[75] Sharpe AH Pauken KE. The Diverse Functions of the PD1 Inhibitory Pathway. Nat Rev Immunol (2018) 18:153–67. 10.1038/nri.2017.108 28990585

[76] Sharpe AH Wherry EJ Ahmed R Freeman GJ. The Function of Programmed Cell Death 1 and Its Ligands in Regulating Autoimmunity and Infection. Nat Immunol (2007) 8:239–45. 10.1038/ni1443 17304234

[77] Liu S Zhang H Li M Hu D Li C Ge B. Recruitment of Grb2 and SHIP1 by the ITT-Like Motif of TIGIT Suppresses Granule Polarization and Cytotoxicity of NK Cells. Cell Death Differ (2013) 20:456–64. 10.1038/cdd.2012.141 PMC3569986 23154388

[78] Zhang B Zhao W Li H Chen Y Tian H Li L. Immunoreceptor TIGIT Inhibits the Cytotoxicity of Human Cytokine-Induced Killer Cells by Interacting With CD155. Cancer Immunol Immunother (2016) 65:305–14. 10.1007/s00262-016-1799-4 PMC11029225 26842126

[79] Yu X Harden K Gonzalez LC Francesco M Chiang E Irving B. The Surface Protein TIGIT Suppresses T Cell Activation by Promoting the Generation of Mature Immunoregulatory Dendritic Cells. Nat Immunol (2009) 10:48–57. 10.1038/ni.1674 19011627

[80] Hutloff A Dittrich AM Beier KC Eljaschewitsch B Kraft R Anagnostopoulos I. ICOS Is an Inducible T-Cell Co-Stimulator Structurally and Functionally Related to CD28. Nature (1999) 397:263–6. 10.1038/16717 9930702

[81] Witsch EJ Peiser M Hutloff A Bchner K Dorner BG Jonuleit H. ICOS and CD28 Reversely Regulate IL-10 on Re-Activation of Human Effector T Cells With Mature Dendritic Cells. Eur J Immunol (2002) 32:2680–6. 10.1002/1521-4141(200209)32:9<2680::AID-IMMU2680>3.0.CO;2-6 12207353

[82] Dong C Juedes AE Temann UA Shresta S Allison JP Ruddle NH. ICOS Co-Stimulatory Receptor Is Essential for T-Cell Activation and Function. Nature (2001) 409:97–101. 10.1038/35051100 11343121

[83] Junger WG. Immune Cell Regulation by Autocrine Purinergic Signalling. Nat Rev Immunol (2011) 11:201–12. 10.1038/nri2938 PMC4209705 21331080

[84] Colgan SP Eltzschig HK Eckle T Thompson LF. Physiological Roles for Ecto-5′-Nucleotidase (CD73). Purinergic Signal (2006) 2:351–60. 10.1007/s11302-005-5302-5 PMC2254482 18404475

[85] Allard B Longhi MS Robson SC Stagg J. The Ectonucleotidases CD39 and CD73: Novel Checkpoint Inhibitor Targets. Immunol Rev (2017) 276:121–44. 10.1111/imr.12528 PMC5338647 28258700

[86] Mandapathil M Lang S Gorelik E Whiteside TL. Isolation of Functional Human Regulatory T Cells (Treg) From the Peripheral Blood Based on the CD39 Expression. J Immunol Methods (2009) 346:55–63. 10.1016/j.jim.2009.05.004 19450601 PMC2703678

[87] Ben Addi A Lefort A Hua X Libert F Communi D Ledent C. Modulation of Murine Dendritic Cell Function by Adenine Nucleotides and Adenosine: Involvement of the A2B Receptor. Eur J Immunol (2008) 38:1610–20. 10.1002/eji.200737781 18465770

[88] Grossman WJ Verbsky JW Tollefsen BL Kemper C Atkinson JP Ley TJ. Differential Expression of Granzymes A and B in Human Cytotoxic Lymphocyte Subsets and T Regulatory Cells. Blood (2004) 104:2840–8. 10.1182/blood-2004-03-0859 15238416

[89] Kawamura K Kadowaki N Kitawaki T Uchiyama T. Virus-Stimulated Plasmacytoid Dendritic Cells Induce CD4+ Cytotoxic Regulatory T Cells. Blood (2006) 107:1031–8. 10.1182/blood-2005-04-1737 16219801

[90] Casares S Hurtado A McEvoy RC Sarukhan A von Boehmer H Brumeanu TD. Down-Regulation of Diabetogenic CD4+ T Cells by a Soluble Dimeric Peptide-MHC Class II Chimera. Nat Immunol (2002) 3:383–91. 10.1038/ni770 11862219

[91] Masteller EL Warner MR Ferlin W Judkowski V Wilson D Glaichenhaus N. Peptide-MHC Class II Dimers as Therapeutics to Modulate Antigen-Specific T Cell Responses in Autoimmune Diabete. J Immunol (2003) 171:5587–95. 10.4049/jimmunol.171.10.5587 14607967

[92] Li L Yi Z Wang B Tisch R. Suppression of Ongoing T Cell-Mediated Autoimmunity by Peptide-MHC Class II Dimer Vaccinatio. J Immunol (2009) 183:4809–16. 10.4049/jimmunol.0901616 PMC5444462 19752238

[93] Grundström S Cederbom L Sundstedt A Scheipers P Ivars F. Superantigen-Induced Regulatory T Cells Display Different Suppressive Functions in the Presence or Absence of Natural CD4 + CD25 + Regulatory T Cells *In Vivo* . J Immunol (2003) 170:5008–17. 10.4049/jimmunol.170.10.5008 12734345

[94] Taylor AL Llewelyn MJ. Superantigen-Induced Proliferation of Human CD4 + CD25 – T Cells Is Followed by a Switch to a Functional Regulatory Phenotyp. J Immunol (2010) 185:6591–8. 10.4049/jimmunol.1002416 21048104

[95] Metzler B Wraith DC. Inhibition of Experimental Autoimmune Encephalomyelitis by Inhalation But Not Oral Administration of the Encephalitogenic Peptide: Influence of MHC Binding Affinity. Int Immunol (1993) 5:1159–65. 10.1093/intimm/5.9.1159 7694644

[96] Gabryšová L Wraith DC. Antigenic Strength Controls the Generation of Antigen-Specific IL-10-Secreting T Regulatory Cells. Eur J Immunol (2010) 40:1386–95. 10.1002/eji.200940151 PMC3466465 20162554

[97] Chang HD Helbig C Tykocinski L Kreher S Koeck J Niesner U. Expression of IL-10 in Th Memory Lymphocytes Is Conditional on IL-12 or IL-4, Unless the IL-10 Gene is Imprinted by GATA-3. Eur J Immunol (2007) 37:807–17. 10.1002/eji.200636385 17304625

[98] Saraiva M Christensen JR Veldhoen M Murphy TL Murphy KM O’Garra A. Interleukin-10 Production by Th1 Cells Requires Interleukin-12-Induced STAT4 Transcription Factor and ERK MAP Kinase Activation by High Antigen Dos. Immunity (2009) 31:209–19. 10.1016/j.immuni.2009.05.012 PMC2791889 19646904

[99] Motomura Y Kitamura H Hijikata A Matsunaga Y Matsumoto K Inoue H. The Transcription Factor E4BP4 Regulates the Production of IL-10 and IL-13 in CD4+ T Cells. Nat Immunol (2011) 12:450–9. 10.1038/ni.2020 PMC3494493 21460847

[100] Levings MK Sangregorio R Galbiati F Squadrone S de Waal Malefyt R Roncarolo M-G. IFN-α and IL-10 Induce the Differentiation of Human Type 1 T Regulatory Cell. J Immunol (2001) 166:5530–9. 10.4049/jimmunol.166.9.5530 11313392

[101] Maynard CL Harrington LE Janowski KM Oliver JR Zindl CL Rudensky AY. Regulatory T Cells Expressing Interleukin 10 Develop From Foxp3+ and Foxp3- Precursor Cells in the Absence of Interleukin 10. Nat Immunol (2007) 8:931–41. 10.1038/ni1504 17694059

[102] Levings MK Gregori S Tresoldi E Cazzaniga S Bonini C Roncarolo MG. Differentiation of Tr1 Cells by Immature Dendritic Cells Requires IL-10 But Not CD25+CD4+ Tr Cells. Blood (2005) 105:1162–9. 10.1182/blood-2004-03-1211 15479730

[103] Shiokawa A Tanabe K Tsuji NM Sato R Hachimura S. IL-10 and IL-27 Producing Dendritic Cells Capable of Enhancing IL-10 Production of T Cells are Induced in Oral Tolerance. Immunol Lett (2009) 125:7–14. 10.1016/j.imlet.2009.05.002 19446579

[104] Comi M Amodio G Gregori S. Interleukin-10-Producing DC-10 Is a Unique Tool to Promote Tolerance Via Antigen-Specific T Regulatory Type 1 Cell. Front Immunol (2018) 9:682. 10.3389/fimmu.2018.00682 29686676 PMC5900789

[105] Pflanz S Timans JC Cheung J Rosales R Kanzler H Gilbert J. IL-27, a Heterodimeric Cytokine Composed of EBI3 and P28 Protein, Induces Proliferation of Naive CD4+T Cells. Immunity (2002) 16:779–90. 10.1016/S1074-7613(02)00324-2 12121660

[106] Murugaiyan G Mittal A Lopez-Diego R Maier LM Anderson DE Weiner HL. IL-27 Is a Key Regulator of IL-10 and IL-17 Production by Human CD4 + T Cell. J Immunol (2009) 183:2435–43. 10.4049/jimmunol.0900568 PMC2904948 19625647

[107] Sun J Dodd H Moser EK Sharma R Braciale TJ. CD4+ T Cell Help and Innate-Derived IL-27 Induce Blimp-1-Dependent IL-10 Production by Antiviral CTL. Nat Immunol (2011) 12:327–35. 10.1038/ni.1996 PMC3079402 21297642

[108] Wang S Miyazaki Y Shinozaki Y Yoshida H. Augmentation of Antigen-Presenting and Th1-Promoting Functions of Dendritic Cells by WSX-1(IL-27r) Deficienc. J Immunol (2007) 179:6421–8. 10.4049/jimmunol.179.10.6421 17982030

[109] Yoshimoto T Yoshimoto T Yasuda K Mizuguchi J Nakanishi K. IL-27 Suppresses Th2 Cell Development and Th2 Cytokines Production From Polarized Th2 Cells: A Novel Therapeutic Way for Th2-Mediated Allergic Inflammatio. J Immunol (2007) 179:4415–23. 10.4049/jimmunol.179.7.4415 17878337

[110] Batten M Li J Yi S Kljavin NM Danilenko DM Lucas S. Interleukin 27 Limits Autoimmune Encephalomyelitis by Suppressing the Development of Interleukin 17-Producing T Cells. Nat Immunol (2006) 7:929–36. 10.1038/ni1375 16906167

[111] Awasthi A Carrier Y Peron JPS Bettelli E Kamanaka M Flavell RA. A Dominant Function for Interleukin 27 in Generating Interleukin 10-Producing Anti-Inflammatory T Cells. Nat Immunol (2007) 8:1380–9. 10.1038/ni1541 17994022

[112] Stumhofer JS Silver JS Laurence A Porrett PM Harris TH Turka LA. Interleukins 27 and 6 Induce STAT3-Mediated T Cell Production of Interleukin 10. Nat Immunol (2007) 8:1363–71. 10.1038/ni1537 17994025

[113] Wang H Meng R Li Z Yang B Liu Y Huang F. IL-27 Induces the Differentiation of Tr1-Like Cells From Human Naive CD4+T Cells *via* the Phosphorylation of STAT1 and STAT3. Immunol Lett (2011) 136:21–8. 10.1016/j.imlet.2010.11.007 21115047

[114] Spolski R Leonard WJ. Interleukin-21: Basic Biology and Implications for Cancer and Autoimmunit. Annu Rev Immunol (2008) 26:57–79. 10.1146/annurev.immunol.26.021607.090316 17953510

[115] Parrish-Novak J Dillon SR Nelson A Hammond A Sprecher C Gross JA. Interleukin 21 and Its Receptor Are Involved in NK Cell Expansion and Regulation of Lymphocyte Function. Nature (2000) 408:57–63. 10.1038/35040504 11081504

[116] Ozaki K Spolski R Feng CG Qi CF Cheng J Sher A. A Critical Role for IL-21 in Regulating Immunoglobulin Production. Science (2002) 298:1630–4. 10.1126/science.1077002 12446913

[117] Ozaki K Spolski R Ettinger R Kim H-P Wang G Qi C-F. Regulation of B Cell Differentiation and Plasma Cell Generation by IL-21, A Novel Inducer of Blimp-1 and Bcl-6. J Immunol (2004) 173:5361–71. 10.4049/jimmunol.173.9.5361 15494482

[118] Vogelzang A McGuire HM Yu D Sprent J Mackay CR King C. A Fundamental Role for Interleukin-21 in the Generation of T Follicular Helper Cell. Immunity (2008) 29:127–37. 10.1016/j.immuni.2008.06.001 18602282

[119] Zeng R Spolski R Finkelstein SE Oh SK Kovanen PE Hinrichs CS. Synergy of IL-21 and IL-15 in Regulating CD8+ T Cell Expansion and Function. J Exp Med (2005) 201:139–48. 10.1084/jem.20041057 PMC2212766 15630141

[120] Jin H Carrio R Yu A Malek TR. Distinct Activation Signals Determine Whether IL-21 Induces B Cell Costimulation, Growth Arrest, or Bim-Dependent Apoptosi. J Immunol (2004) 173:657–65. 10.4049/jimmunol.173.1.657 15210829

[121] Brandt K Bulfone-Paus S Foster DC Rückert R. Interleukin-21 Inhibits Dendritic Cell Activation and Maturation. Blood (2003) 102:4090–8. 10.1182/blood-2003-03-0669 12893770

[122] Spolski R Kim H-P Zhu W Levy DE Leonard WJ. IL-21 Mediates Suppressive Effects *via* Its Induction of IL-10. J Immunol (2009) 182:2859–67. 10.4049/jimmunol.0802978 PMC2756221 19234181

[123] Umeshappa CS Solé P Surewaard BGJ Mohopatra S Yamanouchi J Uddin MM. Liver-Specific T-Regulatory Type 1 Cells Program Local Neutrophils to Suppress Hepatic Autoimmunity *via* Cram. Cell Rep (2021) 34(13):108919. 10.1016/j.celrep.2021.108919 33789099

[124] Greenwald RJ Freeman GJ Sharpe AH. The B7 Family Revisite. Annu Rev Immunol (2005) 23:515–48. 10.1146/annurev.immunol.23.021704.115611 15771580

[125] Nurieva RI Chung Y Hwang D Yang XO Kang HS Ma L. Generation of T Follicular Helper Cells Is Mediated by Interleukin-21 But Independent of T Helper 1, 2, or 17 Cell Lineage. Immunity (2008) 29:138–49. 10.1016/j.immuni.2008.05.009.Generation PMC2556461 18599325

[126] Choi YS Kageyama R Eto D Escobar TC Johnston RJ Monticelli L. ICOS Receptor Instructs T Follicular Helper Cell Versus Effector Cell Differentiation *via* Induction of the Transcriptional Repressor Bcl6. Immunity (2011) 34:932–46. 10.1016/j.immuni.2011.03.023 PMC3124577 21636296

[127] Nurieva RI Duong J Kishikawa H Dianzani U Rojo JM Ho IC. Transcriptional Regulation of Th2 Differentiation by Inducible Costimulator. Immunity (2003) 18:801–11. 10.1016/S1074-7613(03)00144-4 12818161

[128] Serada S Fujimoto M Mihara M Koike N Ohsugi Y Nomura S. IL-6 Blockade Inhibits the Induction of Myelin Antigen-Specific Th17 Cells and Th1 Cells in Experimental Autoimmune Encephalomyelitis. Proc Natl Acad Sci USA (2008) 105:9041–6. 10.1073/pnas.0802218105 PMC2449361 18577591

[129] Fonseca JE Santos MJ Canhão H Choy E. Interleukin-6 as a Key Player in Systemic Inflammation and Joint Destruction. Autoimmun Rev (2009) 8:538–42. 10.1016/j.autrev.2009.01.012 19189867

[130] Lindroos J Svensson L Norsgaard H Lovato P Moller K Hagedorn PH. IL-23-Mediated Epidermal Hyperplasia Is Dependent on IL-6. J Invest Dermatol (2011) 131:1110–8. 10.1038/jid.2010.432 21289639

[131] Hiramatsu Y Suto A Kashiwakuma D Kanari H Kagami S Ikeda K. C-Maf Activates the Promoter and Enhancer of the IL-21 Gene, and TGF-β Inhibits C-Maf-Induced IL-21 Production in CD4 + T Cells. J Leukoc Biol (2010) 87:703–12. 10.1189/jlb.0909639 20042469

[132] Dicosmo BF Picarella D Ravell RA. Local Production of Human IL-6 Promotes Insulitis But Retards the Onset of Insulin-Dependent Diabetes Mellitus in Non-Obese Diabetic Mice. Int Immunol (1994) 6:1829–37. 10.1093/intimm/6.12.1829 7696203

[133] Grivennikov S Karin E Terzic J Mucida D Yu GY Vallabhapurapu S. IL-6 and Stat3 Are Required for Survival of Intestinal Epithelial Cells And Development of Colitis-Associated Cance. Cancer Cell (2009) 15:103–13. 10.1016/j.ccr.2009.01.001 PMC2667107 19185845

[134] Balto K Sasaki H Stashenko P. Interleukin-6 Deficiency Increases Inflammatory Bone Destruction. Infect Immun (2001) 69:744–50. 10.1128/IAI.69.2.744-750.2001 PMC97947 11159963

[135] McGeachy MJ Bak-Jensen KS Chen Y Tato CM Blumenschein W McClanahan T. TGF-β and IL-6 Drive the Production of IL-17 and IL-10 by T Cells and Restrain TH-17 Cell-Mediated Pathology. Nat Immunol (2007) 8:1390–7. 10.1038/ni1539 17994024

[136] Jin JO Han X Yu Q. Interleukin-6 Induces the Generation of IL-10-Producing Tr1 Cells and Suppresses Autoimmune Tissue Inflammation. J Autoimmun (2013) 40:28–44. 10.1016/j.jaut.2012.07.009 22921334 PMC3524403

[137] Novick D Cohen B Rubinstein M. The Human Interferon α β Receptor: Characterization and Molecular Cloning. Cell (1994) 77:391–400. 10.1016/0092-8674(94)90154-6 8181059

[138] Guarda G Braun M Staehli F Tardivel A Mattmann C Förster I. Type I Interferon Inhibits Interleukin-1 Production and Inflammasome Activatio. Immunity (2011) 34:213–23. 10.1016/j.immuni.2011.02.006 21349431

[139] Hu X Paik PK Chen J Yarilina A Kockeritz L Lu TT. IFN-γ Suppresses IL-10 Production and Synergizes With TLR2 by Regulating GSK3 and CREB/AP-1 Protein. Immunity (2006) 24:563–74. 10.1016/j.immuni.2006.02.014 16713974

[140] Garcia CA Benakanakere MR Alard P Kosiewicz MM Kinane DF Martin M. Antigenic Experience Dictates Functional Role of Glycogen Synthase Kinase-3 in Human CD4 + T Cell Response. J Immunol (2008) 181:8363–71. 10.4049/jimmunol.181.12.8363 PMC2849970 19050253

[141] Aman MJ Tretter T Eisenbeis I Bug G Decker T Aulitzky WE. Interferon-Alpha Stimulates Production of Interleukin-10 in Activated CD4+ T Cells and Monocytes. Blood (1996) 87:4731–6. 10.1182/blood.V87.11.4731.bloodjournal87114731 8639843

[142] McRae BL Semnani RT Hayes MP van Seventer GA. Type I IFNs Inhibit Human Dendritic Cell IL-12 Production and Th1 Cell Development. J Immunol (1998) 160:4298–304. 9574532

[143] Ma A Boone DL Lodolce JP. The Pleiotropic Functions of Interleukin 15: Not So Interleukin 2-Like After All. J Exp Med (2000) 191:753–5. 10.1084/jem.191.5.753 PMC2195852 10704456

[144] Waldmann T Tagaya Y Bamford R. Interleukin-2, Interleukin-15, and Their Receptors. Int Rev Immunol (1998) 16:205–26. 10.3109/08830189809042995 9505189

[145] Bulfone-Paus S Ungureanu D Pohl T Lindner G Paus R Rückert R. Interleukin-15 Protects From Lethal Apoptosis *In Vivo* . Nat Med (1997) 3:1124–8. 10.1038/nm1097-1124 9334724

[146] Ku CC Murakami M Sakamoto A Kappler J Marrack P. Control of Homeostasis of CD8+ Memory T Cells by Opposing Cytokines. Science (2000) 288:675–8. 10.1126/science.288.5466.675 10784451

[147] Tough DF Sun S Zhang X Sprent J. Stimulation of Memory T Cells by Cytokines. Vaccine (2000) 18:1642–8. 10.1016/S0264-410X(99)00500-9 10689142

[148] Bacchetta R Sartirana C Levings MK Bordignon C Narula S Roncarolo MG. Growth and Expansion of Human T Regulatory Type 1 Cells Are Independent From TCR Activation But Require Exogenous Cytokines. Eur J Immunol (2002) 32:2237–45. 10.1002/1521-4141(200208)32:8<2237::AID-IMMU2237>3.0.CO;2-2 12209636

[149] Chow KPN Lee JM Qiu JT Liao SK Lin SC Hsu SL. Enhanced IL-10 Production by CD4 + T Cells Primed in IL-15rα-Deficient Mice. Eur J Immunol (2011) 41:3146–56. 10.1002/eji.201141746 21874651

[150] Giri JG Kumaki S Ahdieh M Friend DJ Loomis A Shanebeck K. Identification and Cloning of a Novel IL-15 Binding Protein That Is Structurally Related to the Alpha Chain of the IL-2 Receptor. EMBO J (1995) 14:3654–63. 10.1002/j.1460-2075.1995.tb00035.x PMC394440 7641685

[151] Waldmann TA Tagaya Y. The Multifaced Regulation of Interleukin-15 Expression an the Role of This Cytokine in NK Cell Differentiation and Host Response to Intracellular Pathogens. Annu Rev Immunol (1999) 17:19–49. 10.1146/annurev.immunol.17.1.19 10358752

[152] Papiernik M. Natural CD4+ CD25+ Regulatory T Cells. Their Role in the Control of Superantigen Responses. Immunol Rev (2001) 182:180–9. 10.1034/j.1600-065X.2001.1820114.x 11722633

[153] Anderson PO Sundstedt A Yazici Z Minaee S Woolf R Nicolson K. IL-2 Overcomes the Unresponsiveness But Fails to Reverse the Regulatory Function of Antigen-Induced T Regulatory Cell. J Immunol (2005) 174:310–9. 10.4049/jimmunol.174.1.310 15611254

[154] Tsuji-Takayama K Suzuki M Yamamoto M Harashima A Okochi A Otani T. The Production of IL-10 by Human Regulatory T Cells Is Enhanced by IL-2 Through a STAT5-Responsive Intronic Enhancer in the IL-10 Locu. J Immunol (2008) 181:3897–905. 10.4049/jimmunol.181.6.3897 18768844

[155] Kasprzycka M Zhang Q Witkiewicz A Marzec M Potoczek M Liu X. γc-Signaling Cytokines Induce a Regulatory T Cell Phenotype in Malignant CD4 + T Lymphocyte. J Immunol (2008) 181:2506–12. 10.4049/jimmunol.181.4.2506 PMC2586884 18684941

[156] Andreotti AH Schwartzberg PL Joseph RE Berg LJ. T-Cell Signaling Regulated by the Tec Family Kinase, It. Cold Spring Harb Perspect Biol (2010) 2:1–22. 10.1101/cshperspect.a002287 PMC2890196 20519342

[157] Ahyi A-NN Chang H-C Dent AL Nutt SL Kaplan MH. IFN Regulatory Factor 4 Regulates the Expression of a Subset of Th2 Cytokine. J Immunol (2009) 183:1598–606. 10.4049/jimmunol.0803302 PMC2734910 19592658

[158] Kwon H Thierry-Mieg D Thierry-Mieg J Kim HP Oh J Tunyaplin C. Analysis of Interleukin-21-Induced Prdm1 Gene Regulation Reveals Functional Cooperation of STAT3 and IRF4 Transcription Factor. Immunity (2009) 31:941–52. 10.1016/j.immuni.2009.10.008 PMC3272079 20064451

[159] Cretney E Xin A Shi W Minnich M Masson F Miasari M. The Transcription Factors Blimp-1 and IRF4 Jointly Control the Differentiation and Function of Effector Regulatory T Cells. Nat Immunol (2011) 12:304–12. 10.1038/ni.2006 21378976

[160] Kim JI Ho IC Grusby MJ Glimcher LH. The Transcription Factor C-Maf Controls the Production of Interleukin-4 But Not Other Th2 Cytokines. Immunity (1999) 10:745–51. 10.1016/S1074-7613(00)80073-4 10403649

[161] Zhu J Yamane H Paul WE. Differentiation of Effector CD4 T Cell Population. Annu Rev Immunol (2010) 28:445–89. 10.1146/annurev-immunol-030409-101212 PMC3502616 20192806

[162] Kroenke MA Eto D Locci M Cho M Davidson T Haddad EK. Bcl6 and Maf Cooperate To Instruct Human Follicular Helper CD4 T Cell Differentiatio. J Immunol (2012) 188:3734–44. 10.4049/jimmunol.1103246 PMC3324673 22427637

[163] Harris JE Bishop KD Phillips NE Mordes JP Greiner DL Rossini AA. Early Growth Response Gene-2, a Zinc-Finger Transcription Factor, Is Required for Full Induction of Clonal Anergy in CD4 + T Cell. J Immunol (2004) 173:7331–8. 10.4049/jimmunol.173.12.7331 15585857

[164] Crotty S Johnston RJ Schoenberger SP. Effectors and Memories: Bcl-6 and Blimp-1 in T and B Lymphocyte Differentiation. Nat Immunol (2010) 11:114–20. 10.1038/ni.1837 PMC2864556 20084069

[165] Martins G Calame K. Regulation and Functions of Blimp-1 in T and B Lymphocyte. Annu Rev Immunol (2008) 26:133–69. 10.1146/annurev.immunol.26.021607.090241 18370921

[166] Neumann C Heinrich F Neumann K Junghans V Mashreghi MF Ahlers J. Role of Blimp-1 in Programing Th Effector Cells Into IL-10 Producers. J Exp Med (2014) 211:1807–19. 10.1084/jem.20131548 PMC4144744 25073792

[167] Karwacz K Miraldi ER Pokrovskii M Madi A Yosef N Wortman I. Critical Role of IRF1 and BATF in Forming Chromatin Landscape During Type 1 Regulatory Cell Differentiation. Nat Immunol (2017) 18:412–21. 10.1038/ni.3683 PMC5901650 28166218

[168] Schraml BU Hildner K Ise W Lee WL Smith WAE Solomon B. The AP-1 Transcription Factor Batf Controls T H 17 Differentiation. Nature (2009) 460:405–9. 10.1038/nature08114 PMC2716014 19578362

[169] Ellyard JI Vinuesa CG. A BATF-Ling Connection Between B Cells and Follicular Helper T Cells. Nat Immunol (2011) 12:519–20. 10.1038/ni.2042 21587310

[170] Betz BC Jordan-Williams KL Wang C Kang SG Liao J Logan MR. Batf Coordinates Multiple Aspects of B and T Cell Function Required for Normal Antibody Responses. J Exp Med (2010) 207:933–42. 10.1084/jem.20091548 PMC2867277 20421391

[171] Ciofani M Madar A Galan C Sellars M Mace K Pauli F. A Validated Regulatory Network for Th17 Cell Specification. Cell (2012) 151:289–303. 10.1016/j.cell.2012.09.016 23021777 PMC3503487

[172] Kurachi M Barnitz RA Yosef N Odorizzi PM Diiorio MA Lemieux ME. The Transcription Factor BATF Operates as an Essential Differentiation Checkpoint in Early Effector CD8 + T Cells. Nat Immunol (2014) 15:373–83. 10.1038/ni.2834 PMC4000237 24584090

[173] Ise W Kohyama M Schraml BU Zhang T Schwer B Basu U. The Transcription Factor BATF Controls the Global Regulators of Class-Switch Recombination in Both B Cells and T Cells. Nat Immunol (2011) 12:536–43. 10.1038/ni.2037 PMC3117275 21572431

[174] Zhang P Lee JS Gartlan KH Schuster IS Comerford I Varelias A. Eomesodermin Promotes the Development of Type 1 Regulatory T (TR1) Cells. Sci Immunol (2017) 2. 10.1126/sciimmunol.aah7152 PMC5714294 28738016

[175] Dejean AS Joulia E Walzer T. The Role of Eomes in Human CD4 T Cell Differentiation: A Question of Context. Eur J Immunol (2019) 49:38–41. 10.1002/eji.201848000 30536524

[176] Farez MFF Mascanfroni IDD Méndez-Huergo SPP Yeste A Murugaiyan G Garo LPP. Melatonin Contributes to the Seasonality of Multiple Sclerosis Relapse. Cell (2015) 162:1338–52. 10.1016/j.cell.2015.08.025 PMC4570563 26359987

[177] Im SH Hueber A Monticelli S Kang KH Rao A. Chromatin-Level Regulation of the IL10 Gene in T Cells. J Biol Chem (2004) 279:46818–25. 10.1074/jbc.M401722200 15319439

[178] Miraldi ER Pokrovskii M Watters A Castro DM De Veaux N Hall JA. Leveraging Chromatin Accessibility for Transcriptional Regulatory Network Inference in T Helper 17 Cell. Genome Res (2019) 29:449–63. 10.1101/gr.238253.118 PMC6396413 30696696

[179] Wei L Vahedi G Sun HW Watford WT Takatori H Ramos HL. Discrete Roles of STAT4 and STAT6 Transcription Factors in Tuning Epigenetic Modifications and Transcription During T Helper Cell Differentiation. Immunity (2010) 32:840–51. 10.1016/j.immuni.2010.06.003 PMC2904651 20620946

[180] Li P Spolski R Liao W Wang L Murphy TL Murphy KM. BATF-JUN Is Critical for IRF4-Mediated Transcription in T Cells. Nature (2012) 490:543–6. 10.1038/nature11530 PMC3537508 22992523

[181] Lee CG Hwang W Maeng KE Kwon HK So JS Sahoo A. IRF4 Regulates IL-10 Gene Expression in CD4+ T Cells Through Differential Nuclear Translocation. Cell Immunol (2011) 268:97–104. 10.1016/j.cellimm.2011.02.008 21440248

[182] Vahedi G Takahashi H Nakayamada S Sun HW Sartorelli V Kanno Y. STATs Shape the Active Enhancer Landscape of T Cell Populations. Cell (2012) 151:981–93. 10.1016/j.cell.2012.09.044 PMC3509201 23178119

[183] Shoemaker J Saraiva M O’Garra A. GATA-3 Directly Remodels the IL-10 Locus Independently of IL-4 in CD4 + T Cell. J Immunol (2006) 176:3470–9. 10.4049/jimmunol.176.6.3470 16517715

[184] Samstein RM Arvey A Josefowicz SZ Peng X Reynolds A Sandstrom R. Foxp3 Exploits a Pre-Existent Enhancer Landscape for Regulatory T Cell Lineage Specification. Cell (2012) 151:153–66. 10.1016/j.cell.2012.06.053 PMC3493256 23021222

[185] Hossain DMS Panda AK Manna A Mohanty S Bhattacharjee P Bhattacharyya S. FoxP3 Acts as a Cotranscription Factor With STAT3 in Tumor-Induced Regulatory T Cell. Immunity (2013) 39:1057–69. 10.1016/j.immuni.2013.11.005 24315995

[186] Lee C-G Kwon H-K Sahoo A Hwang W So J-S Hwang J-S. Interaction of Ets-1 With HDAC1 Represses IL-10 Expression in Th1 Cell. J Immunol (2012) 188:2244–53. 10.4049/jimmunol.1101614 22266280

[187] Grenningloh R Bok YK Ho IC. Ets-1, A Functional Cofactor of T-Bet, is Essential for Th1 Inflammatory Responses. J Exp Med (2005) 201:615–26. 10.1084/jem.20041330 PMC2213045 15728239

[188] Lee MT Bonneau AR Giraldez AJ. Zygotic Genome Activation During the Maternal-To-Zygotic Transitio. Annu Rev Cell Dev Biol (2014) 30:581–613. 10.1146/annurev-cellbio-100913-013027 25150012 PMC4303375

[189] Tang WWC Kobayashi T Irie N Dietmann S Surani MA. Specification and Epigenetic Programming of the Human Germ Line. Nat Rev Genet (2016) 17:585–600. 10.1038/nrg.2016.88 27573372

[190] Weinberger L Ayyash M Novershtern N Hanna JH. Dynamic Stem Cell States: Naive to Primed Pluripotency in Rodents and Humans. Nat Rev Mol Cell Biol (2016) 17:155–69. 10.1038/nrm.2015.28 26860365

[191] Hackett JA Azim Surani M. Regulatory Principles of Pluripotency: From the Ground State Up. Cell Stem Cell (2014) 15:416–30. 10.1016/j.stem.2014.09.015 25280218

[192] Smith ZD Meissner A. DNA Methylation: Roles in Mammalian Development. Nat Rev Genet (2013) 14:204–20. 10.1038/nrg3354 23400093

[193] Stergachis AB Neph S Reynolds A Humbert R Miller B Paige SL. Developmental Fate and Cellular Maturity Encoded in Human Regulatory DNA Landscapes. Cell (2013) 154:888–903. 10.1016/j.cell.2013.07.020 23953118 PMC3962256

[194] Ostuni R Piccolo V Barozzi I Polletti S Termanini A Bonifacio S. Latent Enhancers Activated by Stimulation in Differentiated Cells. Cell (2013) 152:157–71. 10.1016/j.cell.2012.12.018 23332752

[195] Ramos-Rodríguez M Raurell-Vila H Colli ML Alvelos MI Subirana-Granés M Juan-Mateu J. The Impact of Proinflammatory Cytokines on the β-Cell Regulatory Landscape Provides Insights Into the Genetics of Type 1 Diabetes. Nat Genet (2019) 51:1588–95. 10.1038/s41588-019-0524-6 PMC7040466 31676868

[196] Onengut-Gumuscu S Chen WM Burren O Cooper NJ Quinlan AR Mychaleckyj JC. Fine Mapping of Type 1 Diabetes Susceptibility Loci and Evidence for Colocalization of Causal Variants With Lymphoid Gene Enhancers. Nat Genet (2015) 47:381–6. 10.1038/ng.3245 PMC4380767 25751624

[197] Farh KKH Marson A Zhu J Kleinewietfeld M Housley WJ Beik S. Genetic and Epigenetic Fine Mapping of Causal Autoimmune Disease Variants. Nature (2015) 518:337–43. 10.1038/nature13835 PMC4336207 25363779

[198] Fasolino M Goldman N Wang W Cattau B Zhou Y Petrovic J. Genetic Variation in Type 1 Diabetes Reconfigures the 3D Chromatin Organization of T Cells and Alters Gene Expressio. Immunity (2020) 52:257–74.e11. 10.1016/j.immuni.2020.01.003 32049053 PMC7152927

[199] Flannick J Florez JC. Type 2 Diabetes: Genetic Data Sharing to Advance Complex Disease Research. Nat Rev Genet (2016) 17:535–49. 10.1038/nrg.2016.56 27402621

[200] Fuchsberger C Flannick J Teslovich TM Mahajan A Agarwala V Gaulton KJ. The Genetic Architecture of Type 2 Diabetes. Nature (2016) 536:41–7. 10.1038/nature18642 PMC5034897 27398621

[201] Whyte WA Orlando DA Hnisz D Abraham BJ Lin CY Kagey MH. Master Transcription Factors and Mediator Establish Super-Enhancers at Key Cell Identity Genes. Cell (2013) 153:307–19. 10.1016/j.cell.2013.03.035 PMC3653129 23582322

[202] Gaulton KJ Nammo T Pasquali L Simon JM Giresi PG Fogarty MP. A Map of Open Chromatin in Human Pancreatic Islets. Nat Genet (2010) 42:255–9. 10.1038/ng.530 PMC2828505 20118932

[203] Pasquali L Gaulton KJ Rodríguez-Seguí SA Mularoni L Miguel-Escalada I Akerman I. Pancreatic Islet Enhancer Clusters Enriched in Type 2 Diabetes Risk-Associated Variants. Nat Genet (2014) 46:136–43. 10.1038/ng.2870 PMC3935450 24413736

[204] Miguel-Escalada I Bonàs-Guarch S Cebola I Ponsa-Cobas J Mendieta-Esteban J Atla G. Human Pancreatic Islet Three-Dimensional Chromatin Architecture Provides Insights Into the Genetics of Type 2 Diabetes. Nat Genet (2019) 51:1137–48. 10.1038/s41588-019-0457-0 PMC6640048 31253982

[205] Sellars M Huh JR Day K Issuree PD Galan C Gobeil S. Regulation of DNA Methylation Dictates Cd4 Expression During the Development of Helper and Cytotoxic T Cell Lineages. Nat Immunol (2015) 16:746–54. 10.1038/ni.3198 PMC4474743 26030024

[206] Bevington SL Cauchy P Piper J Bertrand E Lalli N Jarvis RC. Inducible Chromatin Priming Is Associated With the Establishment of Immunological Memory in T Cells. EMBO J (2016) 35:515–35. 10.15252/embj.201592534 PMC4772849 26796577

[207] Djuretic IM Levanon D Negreanu V Groner Y Rao A Ansel KM. Transcription Factors T-Bet and Runx3 Cooperate to Activate Ifng and Silence Il4 in T Helper Type 1 Cells. Nat Immunol (2007) 8:145–53. 10.1038/ni1424 17195845

[208] Mullen AC Hutchins AS High FA Lee HW Sykes KJ Chodosh LA. Hlx Is Induced by and Genetically Interacts With T-Bet to Promote Heritable THI Gene Induction. Nat Immunol (2002) 3:652–8. 10.1038/ni807 12055627

[209] Usui T Preiss JC Kanno Y Zheng JY Bream JH O’Shea JJ. T-Bet Regulates Th1 Responses Through Essential Effects on GATA-3 Function Rather Than on IFNG Gene Acetylation and Transcription. J Exp Med (2006) 203:755–66. 10.1084/jem.20052165 PMC2118252 16520391

[210] Schoenborn JR Dorschner MO Sekimata M Santer DM Shnyreva M Fitzpatrick DR. Comprehensive Epigenetic Profiling Identifies Multiple Distal Regulatory Elements Directing Transcription of the Gene Encoding Interferon-γ. Nat Immunol (2007) 8:732–42. 10.1038/ni1474 PMC2144744 17546033

[211] Hatton RD Harrington LE Luther RJ Wakefield T Janowski KM Oliver JR. A Distal Conserved Sequence Element Controls Ifng Gene Expression by T Cells and NK Cell. Immunity (2006) 25:717–29. 10.1016/j.immuni.2006.09.007 17070076

[212] Lee DU Avni O Chen L Rao A. A Distal Enhancer in the Interferon-γ (IFN-γ) Locus Revealed by Genome Sequence Comparison. J Biol Chem (2004) 279:4802–10. 10.1074/jbc.M307904200 14607827

[213] Shnyreva M Weaver WM Blanchette M Taylor SL Tompa M Fitzpatrick DR. Evolutionarily Conserved Sequence Elements That Positively Regulate IFN-γ Expression in T Cells. Proc Natl Acad Sci USA (2004) 101:12622–7. 10.1073/pnas.0400849101 PMC515107 15304658

[214] Chen GY Osada H Santamaria-Babi LF Kannagi R. Interaction of GATA-3/T-Bet Transcription Factors Regulates Expression of Sialil Lewis X Homing Receptors on Th1/Th2 Lymphocytes. Proc Natl Acad Sci USA (2006) 103:16894–9. 10.1073/pnas.0607926103 PMC1629005 17075044

[215] Miller SA Huang AC Miazgowicz MM Brassil MM Weinmann AS. Coordinated But Physically Separable Interaction With H3K27-Demethylase and H3K4-Methyltransferase Activities Are Required for T-Box Protein-Mediated Activation of Developmental Gene Expression. Genes Dev (2008) 22:2980–93. 10.1101/gad.1689708 PMC2577798 18981476

[216] Jones B Chen J. Inhibition of IFN-γ Transcription by Site-Specific Methylation During T Helper Cell Development. EMBO J (2006) 25:2443–52. 10.1038/sj.emboj.7601148 PMC1478170 16724115

[217] Chang S Aune TM. Dynamic Changes in Histone-Methylation “Marks” Across the Locus Encoding Interferon-γ During the Differentiation of T Helper Type 2 Cells. Nat Immunol (2007) 8:723–31. 10.1038/ni1473 17546034

[218] Ansel KM Djuretic I Tanasa B Rao A. Regulation of Th2 Differentiation and Il4 Locus Accessibility. Annu Rev Immunol (2006) 24:607–56. 10.1146/annurev.immunol.23.021704.115821 16551261

[219] Tykocinski LO Hajkova P Chang HD Stamm T Sözeri O Löhning M. A Critical Control Element for Interleukin-4 Memory Expression in T Helper Lymphocytes. J Biol Chem (2005) 280:28177–85. 10.1074/jbc.M502038200 15941711

[220] Avni O Lee D Macian F Szabo SJ Glimcher LH Rao A. Th Cell Differentiation is Accompanied by Dynamic Changes in Histone Acetylation of Cytokine Genes. Nat Immunol (2002) 3:643–51. 10.1038/ni808 12055628

[221] Fields PE Kim ST Flavell RA. Cutting Edge: Changes in Histone Acetylation at the IL-4 and IFN-γ Loci Accompany Th1/Th2 Differentiation. J Immunol (2002) 169:647–50. 10.4049/jimmunol.169.2.647 12097365

[222] Koyanagi M Baguet A Martens J Margueron R Jenuwein T Bix M. EZH2 and Histone 3 Trimethyl Lysine 27 Associated With Il4 and Il13 Gene Silencing in TH1 Cells. J Biol Chem (2005) 280:31470–7. 10.1074/jbc.M504766200 16009709

[223] Yamashita M Hirahara K Shinnakasu R Hosokawa H Norikane S Kimura MY. Crucial Role of MLL for the Maintenance of Memory T Helper Type 2 Cell Response. Immunity (2006) 24:611–22. 10.1016/j.immuni.2006.03.017 16713978

[224] Hutchins AS Mullen AC Lee HW Sykes KJ High FA Hendrich BD. Gene Silencing Quantitatively Controls the Function of a Developmental Trans-Activator. Mol Cell (2002) 10:81–91. 10.1016/S1097-2765(02)00564-6 12150909

[225] Makar KW Pérez-Melgosa M Shnyreva M Weaver WM Fitzpatrick DR Wilson CB. Active Recruitment of DNA Methyltransferases Regulates Interleukin 4 in Thymocytes and T Cells. Nat Immunol (2003) 4:1183–90. 10.1038/ni1004 14595437

[226] Wurster AL Pazin MJ. BRG1-Mediated Chromatin Remodeling Regulates Differentiation and Gene Expression of T Helper Cell. Mol Cell Biol (2008) 28:7274–85. 10.1128/mcb.00835-08 PMC2593447 18852284

[227] Ivanov II Zhou L Littman DR. Transcriptional Regulation of Th17 Cell Differentiation. Semin Immunol (2007) 19:409–17. 10.1016/j.smim.2007.10.011 PMC2696342 18053739

[228] McGeachy MJ Cua DJ. Th17 Cell Differentiation: The Long and Winding Roa. Immunity (2008) 28:445–53. 10.1016/j.immuni.2008.03.001 18400187

[229] Zhou L Lopes JE Chong MMW Ivanov II Min R Victora GD. TGF-β-Induced Foxp3 Inhibits TH17 Cell Differentiation by Antagonizing Rorγt Function. Nature (2008) 453:236–40. 10.1038/nature06878 PMC2597437 18368049

[230] Dong C. TH17 Cells in Development: An Updated View of Their Molecular Identity and Genetic Programming. Nat Rev Immunol (2008) 8:337–48. 10.1038/nri2295 18408735

[231] Akimzhanov AM Yang XO Dong C. Chromatin Remodeling of Interleukin-17 (IL-17)-IL-17F Cytokine Gene Locus During Inflammatory Helper T Cell Differentiation. J Biol Chem (2007) 282:5969–72. 10.1074/jbc.C600322200 17218320

[232] Yang XO Pappu BP Nurieva R Akimzhanov A Kang HS Chung Y. T Helper 17 Lineage Differentiation Is Programmed by Orphan Nuclear Receptors Rorα and Rorγ. Immunity (2008) 28:29–39. 10.1016/j.immuni.2007.11.016 18164222 PMC2587175

[233] Johnston RJ Poholek AC DiToro D Yusuf I Eto D Barnett B. Bcl6 and Blimp-1 Are Reciprocal and Antagonistic Regulators of T Follicular Helper Cell Differentiation. Science (2009) 325:1006–10. 10.1126/science.1175870 PMC2766560 19608860

[234] Poholek AC Hansen K Hernandez SG Eto D Chandele A Weinstein JS. In Vivo Regulation of Bcl6 and T Follicular Helper Cell Development. J Immunol (2010) 185:313–26. 10.4049/jimmunol.0904023 PMC2891136 20519643

[235] Lu KT Kanno Y Cannons JL Handon R Bible P Elkahloun AG. Functional and Epigenetic Studies Reveal Multistep Differentiation and Plasticity of In Vitro-Generated and In Vivo-Derived Follicular T Helper Cell. Immunity (2011) 35:622–32. 10.1016/j.immuni.2011.07.015 PMC3235706 22018472

[236] Cannons JL Lu KT Schwartzberg PL. T Follicular Helper Cell Diversity and Plasticity. Trends Immunol (2013) 34:200–7. 10.1016/j.it.2013.01.001 PMC3646926 23395212

[237] Kitagawa Y Ohkura N Kidani Y Vandenbon A Hirota K Kawakami R. Guidance of Regulatory T Cell Development by Satb1-Dependent Super-Enhancer Establishment. Nat Immunol (2017) 18:173–83. 10.1038/ni.3646 PMC5582804 27992401

[238] Toker A Engelbert D Garg G Polansky JK Floess S Miyao T. Active Demethylation of the Foxp3 Locus Leads to the Generation of Stable Regulatory T Cells Within the Thymus. J Immunol (2013) 190:3180–8. 10.4049/jimmunol.1203473 23420886

[239] Ohkura N Hamaguchi M Morikawa H Sugimura K Tanaka A Ito Y. T Cell Receptor Stimulation-Induced Epigenetic Changes and Foxp3 Expression Are Independent and Complementary Events Required for Treg Cell Development. Immunity (2012) 37:785–99. 10.1016/j.immuni.2012.09.010 23123060

[240] Delacher M Imbusch CD Weichenhan D Breiling A Hotz-Wagenblatt A Träger U. Genome-Wide DNA-Methylation Landscape Defines Specialization of Regulatory T Cells in Tissues. Nat Immunol (2017) 18:1160–72. 10.1038/ni.3799 PMC5912503 28783152

[241] Liu B Tahk S Yee KM Fan G Shuai K. The Ligase PIAS1 Restricts Natural Regulatory T Cell Differentiation by Epigenetic Repression. Science (2010) 330:521–5. 10.1126/science.1108297 PMC3043201 20966256

[242] Hegazy AN Peine M Helmstetter C Panse I Fröhlich A Bergthaler A. Interferons Direct Th2 Cell Reprogramming to Generate a Stable GATA-3+T-Bet+ Cell Subset With Combined Th2 and Th1 Cell Function. Immunity (2010) 32:116–28. 10.1016/j.immuni.2009.12.004 20079668

[243] Hirota K Duarte JH Veldhoen M Hornsby E Li Y Cua DJ. Fate Mapping of IL-17-Producing T Cells in Inflammatory Responses. Nat Immunol (2011) 12:255–63. 10.1038/ni.1993 PMC3040235 21278737

[244] Dominguez-Villar M Baecher-Allan CM Hafler DA. Identification of T Helper Type 1-Like, Foxp3+ Regulatory T Cells in Human Autoimmune Disease. Nat Med (2011) 17:673–5. 10.1038/nm.2389 PMC3675886 21540856

[245] Panzer M Sitte S Wirth S Drexler I Sparwasser T Voehringer D. Rapid In Vivo Conversion of Effector T Cells Into Th2 Cells During Helminth Infectio. J Immunol (2012) 188:615–23. 10.4049/jimmunol.1101164 22156341

[246] Schmidl C Hansmann L Andreesen R Edinger M Hoffmann P Rehli M. Epigenetic Reprogramming of the RORC Locus During In Vitro Expansion Is a Distinctive Feature of Human Memory But Not Naïve Tre. Eur J Immunol (2011) 41:1491–8. 10.1002/eji.201041067 21469109

[247] Wei G Wei L Zhu J Zang C Hu-Li J Yao Z. Global Mapping of H3K4me3 and H3K27me3 Reveals Specificity and Plasticity in Lineage Fate Determination of Differentiating CD4+ T Cell. Immunity (2009) 30:155–67. 10.1016/j.immuni.2008.12.009 PMC2722509 19144320

[248] Barnett KR Decato BE Scott TJ Hansen TJ Chen B Attalla J. ATAC-Me Captures Prolonged DNA Methylation of Dynamic Chromatin Accessibility Loci During Cell Fate Transition. Mol Cell (2020) 77:1–15. 10.1016/j.molcel.2020.01.004 31999955 PMC7169048

[249] Kornberg RD Lorch Y. Chromatin Structure and Transcription. Annu Rev Cell Biol (1992) 8:563–87. 10.1146/annurev.cb.08.110192.003023 1335747

[250] Mellor J. The Dynamics of Chromatin Remodeling at Promoters. Mol Cell (2005) 19:147–57. 10.1016/j.molcel.2005.06.023 16039585

